# Artificial Intelligence–Based Prediction of Lung Cancer Risk Using Nonimaging Electronic Medical Records: Deep Learning Approach

**DOI:** 10.2196/26256

**Published:** 2021-08-03

**Authors:** Marvin Chia-Han Yeh, Yu-Hsiang Wang, Hsuan-Chia Yang, Kuan-Jen Bai, Hsiao-Han Wang, Yu-Chuan Jack Li

**Affiliations:** 1 Department of Dermatology Wan Fang Hospital Taipei Medical University Taipei Taiwan; 2 Research Center of Big Data and Meta-analysis Wan Fang Hospital Taipei Medical University Taipei Taiwan; 3 School of Medicine Taipei Medical University Taipei Taiwan; 4 Graduate Institute of Biomedical Informatics College of Medical Science and Technology Taipei Medical University Taipei Taiwan; 5 International Center for Health Information Technology Taipei Medical University Taipei Taiwan; 6 Division of Pulmonary Medicine, Department of Internal Medicine Wan Fang Hospital Taipei Medical University Taipei Taiwan; 7 School of Respiratory Therapy College of Medicine Taipei Medical University Taipei Taiwan; 8 Pulmonary Research Center Wan Fang Hospital Taipei Medical University Taipei Taiwan; 9 Department of Dermatology School of Medicine Taipei Medical University Taipei Taiwan

**Keywords:** artificial intelligence, lung cancer screening, electronic medical record

## Abstract

**Background:**

Artificial intelligence approaches can integrate complex features and can be used to predict a patient’s risk of developing lung cancer, thereby decreasing the need for unnecessary and expensive diagnostic interventions.

**Objective:**

The aim of this study was to use electronic medical records to prescreen patients who are at risk of developing lung cancer.

**Methods:**

We randomly selected 2 million participants from the Taiwan National Health Insurance Research Database who received care between 1999 and 2013. We built a predictive lung cancer screening model with neural networks that were trained and validated using pre-2012 data, and we tested the model prospectively on post-2012 data. An age- and gender-matched subgroup that was 10 times larger than the original lung cancer group was used to assess the predictive power of the electronic medical record. Discrimination (area under the receiver operating characteristic curve [AUC]) and calibration analyses were performed.

**Results:**

The analysis included 11,617 patients with lung cancer and 1,423,154 control patients. The model achieved AUCs of 0.90 for the overall population and 0.87 in patients ≥55 years of age. The AUC in the matched subgroup was 0.82. The positive predictive value was highest (14.3%) among people aged ≥55 years with a pre-existing history of lung disease.

**Conclusions:**

Our model achieved excellent performance in predicting lung cancer within 1 year and has potential to be deployed for digital patient screening. Convolution neural networks facilitate the effective use of EMRs to identify individuals at high risk for developing lung cancer.

## Introduction

Lung cancer is a leading cause of cancer death worldwide, and to reduce its mortality, early detection is crucial. The National Lung Cancer Screening Trial (NLST) revealed that screening with low-dose computed tomography (LDCT) can reduce the mortality associated with lung cancer by 20% [1]. Likewise, the Dutch-Belgian Randomized Lung Cancer Screening Trial (NELSON study) recently revealed that screening with LDCT resulted in a 24% decrease in the 10-year cumulative mortality for men and a 33% decrease for women [2]. Multiple organizations have recommended LDCT screening for lung cancer to be used on target populations [3,4]. Given the potential harm due to radiation exposure, false-positive results, and costs associated with LDCT, most organizations only recommend annual screening that targets high-risk individuals; this group is largely identified by epidemiological factors, including age and smoking/cessation history [5]. Furthermore, due to the potential harm associated with false-positive results, the cost-effectiveness of implementing annual LDCT screening remains controversial [6]. Multiple research groups have attempted to overcome this problem by developing risk prediction models to identify patients who might benefit from LDCT screening and to generate criteria that are superior to those introduced by the NLST and related studies [7-14]. These models frequently include self-reported information, such as family history, BMI, socioeconomic status, and smoking/cessation history, and they use conventional regression models for the final risk analysis.

In the era of digital medicine, the use of artificial intelligence has resulted in good performance for predicting image-related tasks, specifically the use of convolutional neural networks (CNNs). In lung cancer research, CNNs have been applied to LDCT and chest radiographic images to facilitate detection and classification of pulmonary nodules; these models demonstrate performance that is comparable to that achieved by human experts [15-19]. The prediction performance is largely based on high-level feature extraction and nonlinear prediction via the use of CNNs. Given proper data conversion, using CNN methodologies to generate predictions using other nonimaging medical data may be possible. Our group recently described a risk prediction model for nonmelanoma skin cancer that was generated using data extracted from electronic medical records (EMRs) [20].

In predicting lung cancer risk, the EMR should be suited to the task of identifying high-risk individuals [21]. In this study, our goal is to develop a risk model for the prediction of lung cancer using data from EMRs. As such, we applied established CNN algorithms to the large data set available in EMRs to identify important patterns associated with the development of lung cancer. In contrast with methods used for traditional regression analysis, we attempted to include evolving sequential information found in EMRs to generate our prediction model. Our goal was to generate a model that facilitated the prospective identification of individuals at higher risk for developing lung cancer; these individuals might then undergo further follow-up examinations, including LDCT. The use of a predictive model to identify individuals at high risk could serve to limit unnecessary radiation exposure and reduce costs associated with LDCT and related interventions.

## Methods

### Study Population

Deidentified EMRs of 2 million patients who received care between January 01, 1999, and December 31, 2013, were initially sampled from the Taiwan National Health Insurance Research Database (NHIRD). These EMRs included the demographic information, diagnoses, and procedure codes from the *International Classification of Diseases, Ninth Revision, Clinical Modification* (*ICD-9-CM*) and prescriptions from both outpatient clinical declaration files and in-hospital declaration files. This study included participants between the ages of 20 and 90 years who had at least 4 years of medical records on file. Participants with missing data were excluded. These criteria yielded 1,628,250 EMRs with over 300 million record entries for evaluation and analysis. This study was approved by the Taipei Medical University Institutional Review Board; informed patient consent was waived, as all data were anonymous and deidentified before analysis [22].

### Data Preprocessing

**Figure 1:**
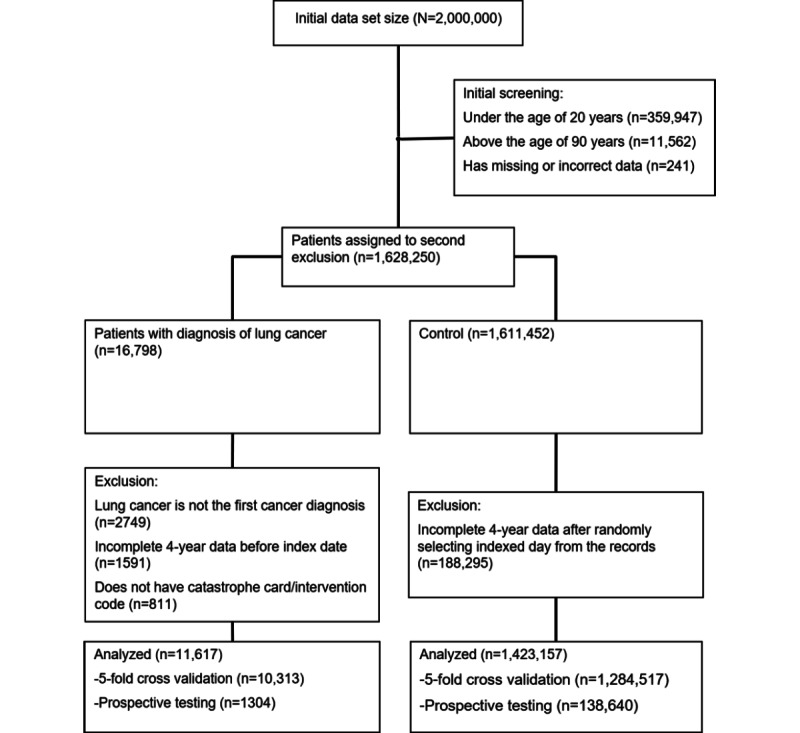
Inclusion and exclusion criteria for the study.

Previous validation studies that focused on lung cancer using the NHIRD have shown a positive predictive value (PPV) of 95% [23]. In this study, we provide further validation of the diagnosis of lung cancer using intervention codes (eg, thoracic surgery, subsequent radiotherapy, or chemotherapy) and national catastrophic illness cards (which require definite pathologic proof of a cancer diagnosis). The inclusion and exclusion criteria used in this study are indicated in Figure 1.

**Figure 2:**
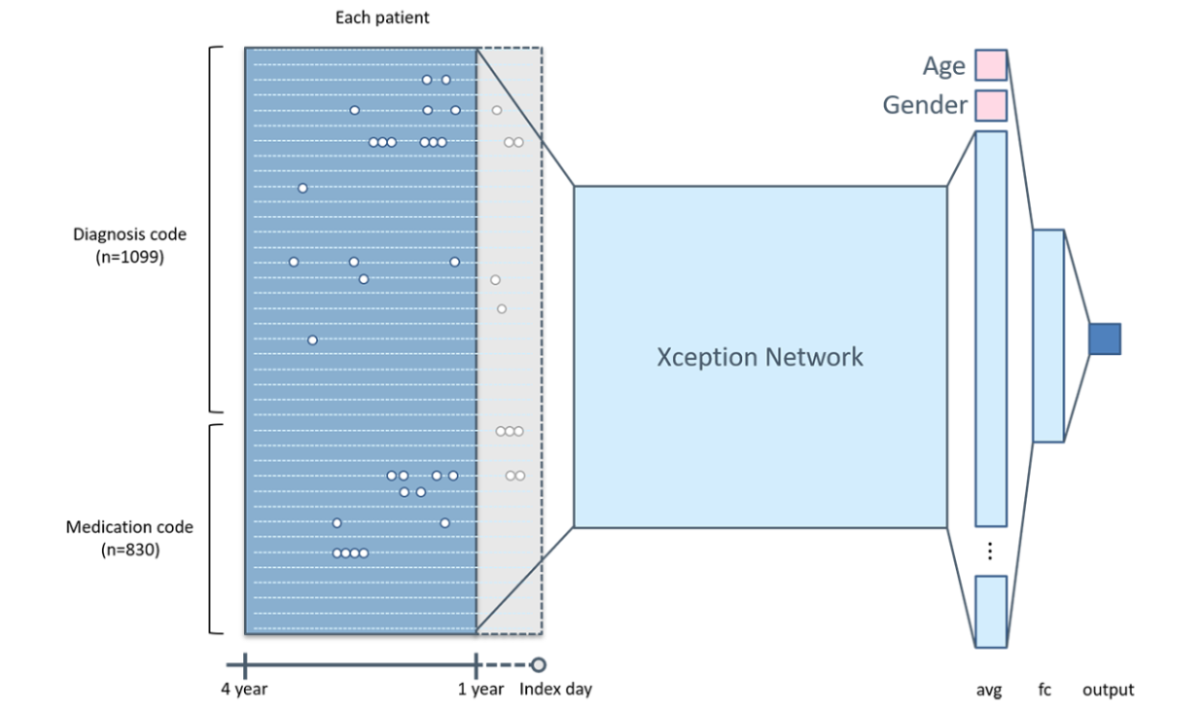
Visualization of the hidden layer of the model using t-stochastic neighbor embedding. Avg: average; fc: fully connected layer.

The index date for patients with lung cancer was defined as the date of first diagnosis. For the control patients, the index dates were randomly selected from their medical history. *ICD-9-CM* diagnosis codes and World Health Organization-Anatomical Therapeutic Chemical (WHO-ATC) prescription codes were collected from each case for preprocessing; the date 1 year prior to the index date was used to define the prediction window. The observation window included the 3 years prior to the date included in the prediction window. Thus, we used 3 years of patient medical information to predict the risk of new-onset lung cancer at or within 1 year later (Figure 2). The *ICD-9-CM* and WHO-ATC codes were preprocessed as described in our previous study [20]. Briefly, the EMRs were classified into 1099 *ICD-9-CM* code groups and 830 WHO-ATC drug groups. Together, 1929 features were recorded weekly for 157 weeks. For each patient, the diagnoses and medications prescribed at each visit were recorded and converted to an image-like array that preserved temporal information associated with both diagnosis and medication history.

The inputs included age, gender, and an image representing the patient’s 3-year history of diagnosis and medication. The image was input into Xception, a 126-layer neural network, in which feature extraction was performed. The final layer of the Xception network was connected to an average pooling layer and then connected to a fully connected layer with the patient’s age and gender.

We performed 3 subgroup analyses to investigate the performance of the model in different populations. According to the age criteria used in previous trials focused on lung cancer screening [1], patients above and below 55 years of age were included among the subgroups. We also examined patients both with and without previous lung disease [24], including subgroups of patients diagnosed with asbestosis, bronchiectasis, chronic bronchitis, chronic obstructive pulmonary disease (COPD), emphysema, fibrosis, pneumonia, sarcoidosis, silicosis, and tuberculosis. Finally, to focus on the discriminative power of the diagnosis and medication without the confounding effects of age, a subgroup of age- and gender-matched controls was identified.

### Model Construction and Evaluation

All patient data were split into training, validation, and testing sets based on their respective index dates. Data with index dates prior to December 31, 2012, were used for training and internal validation, and data with index dates after that date were used for prospective testing. The patients’ age, gender, and image-like arrays described above were used as inputs to generate the model (Figure 2).

Lung cancer risk prediction was treated as a binary classification task using supervised learning. The model was trained to determine whether a given patient was likely to develop lung cancer within 1 year. The Xception architecture [25], which includes a 126-layer CNN-based neural network with a moderate number of parameters, was used for feature extraction. The detailed model structure is shown in Figure 2; the model construction and hyperparameters are listed in Section S1 in Multimedia Appendix 1. During training, class weights based on the population size were set to address data imbalance. To ensure the robustness of the model, a 5-fold cross validation was performed on the model. The performance of the model was assessed by its sensitivity, specificity, and area under the receiver operating characteristic curve (AUC). Model calibration was assessed using a reliability curve and the median absolute error.

To understand the model prediction, occlusion sensitivity analysis was performed by iteratively masking information from a single diagnosis or medication followed by evaluating any changes in the model prediction [26]. In addition, a dimensional reduction technique, t-distributed stochastic neighbor embedding (t-SNE), was performed on the fully connected hidden layer output of the final testing data. We randomly selected 1000 lung cancer patients and 9000 control patients for visualization. The model construction, data preprocessing, model training, and statistical processing were performed using the Python programming language, version 3.6.

## Results

### Baseline Demographics

A total of 11,617 lung cancer patients and 1,423,154 control patients were identified in our data set. The mean age of the lung cancer group was 66.62 years (SD 14.01); the overall data set included 856,558 (59.7%) men and 578,213 (40.3%) women. The baseline demographics of this patient cohort and the assigned subgroups are summarized in Table 1 and Tables S1-S10 in Multimedia Appendix 1. 

**Table 1:** Demographics of the patients with lung cancer and control patients (N=1,434,771).

Group		Patients, n	Age (years), mean (SD)	Male gender, n (%)	Mean diagnosis record count (SD), n	Mean medication record count (SD), n	
**Whole population**							
	Lung cancer	11,617	66.62 (14.01)	6931 (59.7)	121.62 (113.19)	202.68 (208.97)	
	Control	1,423,154	44.95 (16.32)	683,375 (48.0)	66.09 (76.60)	105.99 (135.54)	
**Age and gender match (1:10)**							
	Lung cancer	11,617	66.62 (14.01)	6931 (59.7)	121.62 (113.19)	202.68 (208.97)	
	Control	116,169	66.62 (14.01)	69,310 (59.7)	117.99 (113.67)	190.22 (196.78)	
**Age ≥55 years**							
	Lung cancer	9261	71.99 (9.46)	5673 (61.3)	135.12 (116.31)	227.81 (218.12)	
	Control	385,052	66.57 (9.04)	56,730 (48.6)	114.23 (106.76)	184.50 (189.50)	
**Age <55 years**							
	Lung cancer	2356	45.50 (7.55)	1258 (53.4)	68.58 (80.42)	103.90 (126.71)	
	Control	1,038,102	36.93 (9.85)	496,256 (47.8)	48.23 (51.36)	76.87 (93.45)	
**History of lung disease^a^**							
	Lung cancer	3565	70.79 (12.73)	2244 (63.0)	175.12 (134.36)	297.56 (245.55)	
	Control	182,098	53.01 (18.09)	85,070(46.7)	125.17 (114.53)	204.85 (204.66)	
**No history of lung disease**							
	Lung cancer	8052	64.77 (14.16)	4687 (58.2)	97.94 (93.08)	160.67 (174.80)	
	Control	1,270,651	43.77 (15.70)	598,305 (48.2)	57.42 (64.94)	91.48 (115.23)	

^a^Lung diseases included asbestosis, bronchiectasis, chronic bronchitis, chronic obstructive pulmonary disease, emphysema, fibrosis, pneumonia, sarcoidosis, silicosis, and tuberculosis. More information is provided in Table S11 in Multimedia Appendix 1.

### Model Performance

For all patients, the model revealed an AUC of 0.821 when the input image-like array included sequential diagnostic information only. By contrast, the AUC was 0.894 when the input features included sequential medication information only; when the sequential diagnostic and medication information was simplified to binary variables, the model performance decreased (AUC=0.827). When both sequential diagnostic and medication information were integrated, the model reached an AUC of 0.902 on prospective testing, with a sensitivity of 0.804 and specificity of 0.837 (Table S12 in Multimedia Appendix 1). The calibration of the model showed a median expected error of 0.125; the reliability curve is shown in Figure S1 in Multimedia Appendix 1.

The model performance at different age cutoffs was then investigated. Screening using an age cutoff of 55 years revealed a superior AUC of 0.871 compared to those obtained when cutoffs of 50 or 60 years were used (0.866 and 0.863, respectively) (Table S13, Multimedia Appendix 1).

### Subgroup Analysis

Analyses of the subgroups included one that was both age- and-gender-matched, those at ages above and below 55 years, and those with or without lung disease were performed. For this analysis, we identified an age- and gender-matched control subgroup that was 10 times larger than the original lung cancer subgroup. This model revealed an AUC of 0.818 (SD 0.005) with a sensitivity of 0.647 (SD 0.017) and a specificity of 0.873 (0.023 SD), as shown in Table 2 and in Table S14 in Multimedia Appendix 1. For patients above 55 years of age, the model revealed an AUC of 0.869 (SD 0.005) with a sensitivity of 0.784 (SD 0.011) and a specificity of 0.785 (SD 0.016). The PPV in this subgroup was 0.081% (SD 0.005%), and the negative predictive value was 0.993% (SD 0.000%). The performance of the model was inferior in patients below the age of 55 years; however, it still achieved an AUC of 0.815 (SD 0.007). The discriminatory powers of these models were both excellent among patients with and without a history of lung disease; the AUCs for these subgroups were 0.914 (SD 0.003) and 0.887 (SD 0.002), respectively. Among all the subgroups, the model had the weakest performance in patients below 55 years of age who had no history of lung disease; the AUC for this subgroup was only 0.797 (SD 0.008) for the one-year prospective prediction. By contrast, the model provided the strongest performance for individuals above the age of 55 years with a history of lung disease, which revealed the highest PPV of 14.3% (SD 2.3%). The model exhibited the lowest PPV of 1.0% (SD 0.2%) for individuals less than 55 years of age with no history of lung disease (Table 2). The receiver operating characteristic curves associated with each of these subgroups are summarized in sections S2.1-S2.9 in Multimedia Appendix 1.

**Table 2:** Discrimination performance (testing set) of the model in the subgroups.

Subgroup	Lung cancer group, n	Control, n	Testing AUC^a^ (SD)	Testing sensitivity (SD)	Testing specificity (SD)	PPV^b^ (SD), %	NPV^c^ (SD), %
Whole population	1304	138,640	0.898 (0.002)	0.805 (0.015)	0.825 (0.018)	4.2 (0.3)	99.8 (0)
Matching age and gender	1304	13,040	0.818 (0.005)	0.647 (0.017)	0.873 (0.023)	34.6 (0.4)	96.0 (0.1)
Age ≥55 years	1046	43,328	0.869 (0.002)	0.784 (0.011)	0.785 (0.016)	8.1 (0.5)	99.3 (0)
Age <55 years	258	95,312	0.815 (0.007)	0.620 (0.080)	0.838 (0.054)	1.1 (0.2)	99.9 (0)
History of lung disease	361	16,596	*0.914* (*0.003*)^d^	0.829 (0.021)	0.816 (0.021)	9.0 (0.8)	0.995 (0.1)
No history of lung disease	943	122,044	0.887 (0.002)	0.781 (0.025)	0.827 (0.026)	3.4 (0.5)	99.8 (0.0)
Age ≥55 years with history of lung disease	318	8184	0.875 (0.005)	0.755 (0.047)	0.819 (0.044)	*14.3* (*2.3*)	98.9 (0.2)
Age ≥55 years with no history of lung disease	728	35,144	0.865 (0.003)	0.775 (0.019)	0.786 (0.018)	7.0 (0.4)	99.4 (0.0)
Age <55 years with history of lung disease	43	8,412	0.909 (0.006)	0.777 (0.054)	0.891 (0.036)	3.8 (1.0)	99.9 (0.0)
Age <55 years with no history of lung disease	215	86,900	0.797 (0.008)	0.533 (0.048)	0.865 (0.026)	*1.0* (*0.2*)	99.9 (0.0)

^a^AUC: area under the curve.

^b^PPV: positive predictive value.

^c^NPV: negative predictive value.

^d^Italic text indicates the best performance for the parameter.

**Figure 3:**
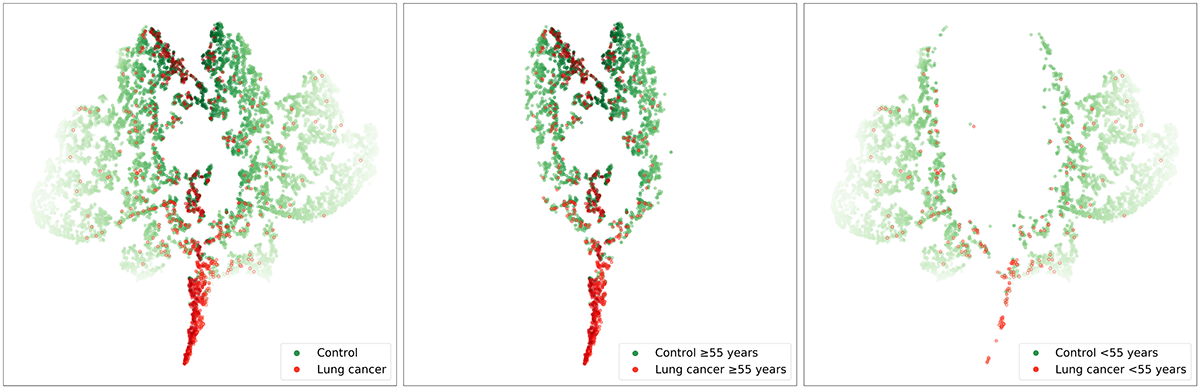
Visualization of the hidden layer of the model using t-stochastic neighbor embedding.

Table 3 summarizes the age, gender, diagnosis, and medications associated with both the correctly and incorrectly classified groups from the testing data set. The mean age of the true-positive group was similar to that of the false-positive group and somewhat greater than that of the false-negative group. This tendency was also observed in other subgroups; overall, our results suggest that age and sex are important predictive factors. This is consistent with the t-SNE analysis, in which patients with lung cancer and control patients over 55 years of age were clustered centrally, as compared to the other patients, who were located at the periphery (Figure 3).

The model’s hidden layer outputs of 1000 patients with cancer (red dots) and 9000 control patients (green dots) were visualized using t-SNE (Figure 3). Dark green and red represent old age control patients and patients with cancer, respectively. As shown in the left image, most patients with cancer can be clustered away from the control patients. Some dark red dots are mixed with dark green dots in the upper area. These are the patients that were wrongly predicted to be controls by the model. The center images shows that patients aged ≥55 years were clustered in the center of the graph, with the patients with cancer were successfully clustered in the tip area. The right image shows that patients aged <55 years were clustered at the periphery of the graph. Some patients with cancer were also clustered in the tip area, whereas the others were scattered with the control patients.

Occlusion sensitivity analysis further revealed that the specific diagnosis and medication factors were associated with an increased risk of developing lung cancer. Interestingly, “other noninfectious gastroenteritis and colitis” and “other agents for local oral treatment” were associated with the highest risks of developing lung cancer with respect to patient diagnosis and medication, respectively. The top 20 factors identified in the analysis are summarized in Table 4.

**Table 3:** Prediction analysis of the prospective testing data set (N=139,944).

Group		Patients, n	Age (years), mean (SD)	Male gender, n (%)	Mean diagnosis count (SD), n	Mean medication count (SD), n
**All patients**						
	True positive	1052	69.91 (11.58)	617 (58.65)	141.75 (113.31)	210.7 (186.32)
	False positive	22,624	69.19 (12.48)	12,641 (55.87)	114.96 (111.04)	159.14 (171.74)
	True negative	116,016	41.94 (13.14)	53,671 (46.26)	63.08 (67.53)	81.46 (101.84)
	False negative	252	50.96 (10.79)	134 (53.17)	81.37 (95.67)	104.03 (139.98)
**Patients aged ≥55 years**						
	True positive	851	72.86 (9.25)	510 (59.93)	146.32 (110.84)	217.88 (181.04)
	False positive	10,989	74.88 (9.66)	6640 (60.42)	124.11 (119.27)	170.8 (179.15)
	True negative	32,339	63.28 (6.58)	13,871 (42.89)	110.24 (97.26)	152.69 (154.96)
	False negative	195	64.62 (6.63)	106 (54.36)	125.98 (132.09)	185.08 (216.55)
**Patients aged <55 years**						
	True positive	209	47.87 (6.07)	113 (54.07)	83.3 (87.98)	106.48 (128.64)
	False positive	32,765	46.78 (6.58)	18,422 (56.22)	59.4 (63.22)	74.38 (92.27)
	True negative	62,547	32.45 (7.43)	27,379 (43.77)	48.67 (48.88)	60.74 (71.36)
	False negative	49	36.22 (5.82)	22 (44.90)	63.98 (63.75)	83.88 (115.66)
**Patients with a history of lung disease**						
	True positive	300	72.86 (11.18)	182 (60.67)	184.91 (118.07)	278.71 (194.81)
	False positive	2791	75.41 (11.97)	1750 (62.70)	180.66 (140.56)	253.68 (214.05)
	True negative	13,805	49.34 (15.6)	5876 (42.56)	119.33 (102.8)	162.24 (162.85)
	False negative	61	61.41 (12.11)	34(55.74)	171.72 (155.81)	246.79 (226.86)
**Patients with no history of lung disease**						
	True positive	757	68.45 (11.4)	442 (58.39)	120.97 (104.28)	177.03 (172.5)
	False positive	23,328	66.54 (12.25)	12,881 (55.22)	95.23 (94.24)	130.24 (146.34)
	True negative	98,716	40.39 (12.27)	45,805 (46.40)	56.19 (59.51)	71.56 (88.63)
	False negative	186	48.19 (10.32)	93 (50.00)	65.08 (66.98)	81.69 (101.83)
**Patients aged ≥55 years with a history of lung disease**						
	True positive	255	74.89 (9.03)	160 (62.75)	188.33 (119.58)	284.4 (193.99)
	False positive	1778	78.53 (9.16)	1205 (67.77)	188.16 (142.99)	263 (215.97)
	True negative	6406	66.38 (7.88)	2669 (41.66)	169.82 (121.41)	239.26 (195.71)
	False negative	63	70.44 (7.81)	35 (55.56)	203.87 (148.87)	308.17 (221.29)
**Patients aged ≥55 years with no history of lung disease**						
	True positive	587	71.76 (9.24)	347(59.11)	126.04 (102.89)	185.01 (166.72)
	False positive	8958	73.86 (9.69)	5,281(58.95)	104.85 (103.3)	142.56 (154.72)
	True negative	26,186	62.73 (6.27)	11,356(43.37)	98.04 (87.47)	135.09 (139.76)
	False negative	141	63.47 (6.25)	74(52.48)	100.89 (103.77)	148.73 (195.18)
**Patients aged <55 years with lung diseases**						
	True positive	37	48.89 (6.08)	18 (48.65)	120.46 (100.27)	157.62 (173.25)
	False positive	1080	46.56 (7.56)	653 (60.46)	85.56 (72.24)	109.78 (108.74)
	True negative	7332	37.7 (9.58)	3099 (42.27)	86.84 (75.16)	113.06 (116.51)
	False negative	6	43.33 (9.24)	3 (50.00)	103.67 (98.36)	149.83 (152.85)
**Patients aged <55 years with no history of lung disease**						
	True positive	172	47.55 (6.07)	95(55.23)	74.94 (83.33)	94.44 (114.72)
	False positive	30,982	46.56 (6.56)	17,478(56.41)	55.1 (58.63)	68.47 (84.96)
	True negative	55,918	32.06 (7.25)	24,571(43.94)	45.68 (45.68)	56.64 (65.81)
	False negative	43	35.65 (5.54)	19(44.19)	59.88 (56.98)	78.84 (108.63)

**Table 4:** Top 20 factors related to lung cancer learned by the model.

Rank	Factor	Lung cancer risk increase (%), mean (SD)
1	Other noninfectious gastroenteritis and colitis	1.85 (1.01)
2	Other congenital anomalies of the circulatory system	1.84 (2.21)
3	Other agents for local oral treatment	1.76 (1.02)
4	Antidotes	1.69 (1.55)
5	Postinflammatory pulmonary fibrosis	1.69 (1.43)
6	Metronidazole	1.69 (1.29)
7	Acariasis	1.65 (1.73)
8	Antiviral drugs	1.57 (1.03)
9	Orchitis and epididymitis	1.57 (1.48)
10	Pneumococcal pneumonia	1.52 (0.93)
11	Buflomedil	1.44 (1.76)
12	Danazol	1.42 (1.41)
13	Calcineurin inhibitors	1.42 (1.29)
14	Other disorders of the urethra and urinary tract	1.37 (1.34)
15	Angina pectoris	1.35 (1.44)
16	Other nonorganic psychoses	1.35 (1.99)
17	Respiratory conditions due to other and unspecified external agents	1.33 (1.33)
18	Open wound of back	1.33 (2.46)
19	Hydrazinophthalazine derivatives	1.31 (1.57)
20	Insulin	1.30 (1.51)

## Discussion

### Principal Findings

In this study, we explored the possibility of predicting lung cancer using a CNN with diagnosis and medication history extracted from EMRs as a data source. Unlike other proposed lung cancer risk models, our model does not rely on self-reported parameters such as smoking/cessation history, family history, socioeconomic status, or BMI. This model could be readily deployed as a means to evaluate centralized health care databases and perform efficient population-based screening. Such an approach has potential to improve the accuracy of current screening methods, as it can identify those most likely to benefit from interventions [21]. In addition, we attempted to include time-related sequential information as reflected in the medical histories as a means to evaluate lung cancer risk. This approach is different from those used in traditional regression analysis, in which personal history is often simplified and limited to binary or categorical variables. We found that the integration of temporal aspects resulted in improvements in the model performance (Table S12 in Multimedia Appendix 1). The capacity for complex integration of multiple variables is one of the strengths of deep neural networks. To generate this model, we used an established computer vision model (Xception) to extract high-level features from the array representing individual clinical case histories; this ensured that the high-level features associated with the clinical information were effectively extracted for risk prediction.

### Related Work

Lung cancer prediction models are under investigation with the goal of identifying high-risk populations that might benefit from LDCT screening. A variety of parameters have been used for prediction, including epidemiologic factors (eg, socioeconomic status, BMI, and smoking history), clinical history (eg, family history and individual history of lung disease history), and results of clinical examinations (eg, blood tests, genetic analysis, and imaging results). The PLCOm2012 model is the most widely validated, with AUCs of 0.78 to 0.82 [27-30]. Likewise, the Bach model exhibited AUCs of 0.66 to 0.75 on external validation [5,31]. Other models include the Haggart model, which exhibited AUCs of 0.71 to 0.84 [5,9], the Liverpool Lung Project model, with AUCs of 0.67 to 0.82 [32], and the Lung Cancer Risk Assessment Tool, which achieved AUCs of 0.77 to 0.78 [5,33]. Some models used information extracted from patient EMRs. The model proposed by Iyen-Omofoman et al [10] used lung-associated clinical symptoms and social-epidemiologic factors from a general practice database, and they achieved an AUC of 0.88; likewise, Wang et al [13] included 33,788 clinical features from clinical histories and laboratory tests evaluated in an extreme gradient boosting (XGBoost) model to achieve an AUC of 0.88. With these previous studies in mind, our model featured a deep learning approach and achieved a prospective prediction AUC of 0.87 in patients older than 55 years and 0.90 for the entire patient cohort. It is possible to test other machine learning models (eg, support vector machine or random forest) on our data set. However, this study serves as a proof of concept of using CNN with nonimaging medical records. Comparing the performance of this model to that of different machine learning models of practical interest would be an interesting approach for future studies.

We recognize that direct comparisons between models may not be fully appropriate, as the target populations and predicted outcomes can vary. Previous reports suggested that the performance of models is inflated when nonsmokers and younger subjects (<55 years of age) are included in the study groups [34]. Our findings confirm this point, as can be observed from the higher AUCs associated with the younger age cutoffs (Table S3, Multimedia Appendix 1). Although our data set did not directly include reports of smoking history or cessation, we did include a history of lung diseases (eg, chronic bronchitis, COPD, and emphysema) among our parameters; these could easily be considered as surrogate factors for smoking history. Further analysis of this patient subgroup may help us understand and mitigate the possibility of performance inflation.

In the original NLST trial, the PPV for the LDCT was determined to be 3.4% [1]. The high false-positive rate associated with this intervention remains a major concern with respect to LDCT screening. In this study, the highest PPV (14.5%) was observed in patients ≥55 years of age with a history of lung disease. As noted above, an increase in cancer diagnoses might be expected in this patient subgroup, as a history of lung disease may be a direct consequence of smoking. As such, this finding suggested that individuals in this subgroup are suitable candidates for model prescreening in an effort to avoid unnecessary radiation exposure and costs associated with LDCT. In addition, we found that the 55-year age cutoff selected in the original NLST trial was also appropriate for our model, as the predictive performance was higher with this age cutoff compared to that observed at cutoffs at age 50 or 60 years (Table S3, Multimedia Appendix 1).

### Predictive Factor Analysis

The inclusion of an age- and gender-matched subgroup was necessary to explore the roles of clinical diagnosis and medication history in the predictions generated by our model; evaluation of this subgroup prevented the confounding effects of age and its correlations to clinical history (eg, older people are typically prescribed more chronic disease-related medications). With this consideration, our model achieved an AUC of 0.818. These findings can be compared to the model proposed by Spitz et al [12], which included gender-, age-, and smoking status–matched patients and achieved an AUC of 0.63 in former smokers. Although the models generated from matched populations tended to display weaker performance than those from nonmatched populations and may not be clinically useful, this result provided us with a more clear-cut evaluation of the specific parameters included in this model. Taken together, our findings suggest that our model is capable of identifying factors that are useful for predicting lung cancer using clinical information available 1 year before the clinical diagnosis is made.

Our model demonstrated the worst performance in young patients without pre-existing lung diseases. This finding suggests that identifying high-risk patients among young and asymptomatic patients is still the most challenging task. Further studies are required to assess the performance of the model in patients with different staging. One of the major concerns with respect to the use of lung cancer prediction models is that they tend to select individuals who are older and who have multiple comorbidities [35], thus reducing the overall benefit gained from the screening process [36]. This tendency was also observed in our model. This phenomenon cannot be fully avoided, as it simply reflects the high percentage of older patients in the population who are diagnosed with lung cancer. However, when focused on patients younger than 55 years of age, our model maintained excellent discriminative power (the AUC was 0.82, with a mean age of true positives of 47.8 years). With the current model, the inclusion of younger individuals remains possible; multiple age-stratified thresholds for lung cancer risk could further optimize the clinical benefits of the predictions from this model.

Although deep learning is often considered a “black box,” and it is often challenging to explain the reasoning behind the outcomes, our study used t-SNE and occlusion sensitivity analysis to identify the most critical of the contributing parameters. Our occlusion sensitivity analysis revealed that many of the important factors were those associated with a history of preexisting lung conditions (eg, postinflammatory pulmonary fibrosis and pneumococcal pneumonia) and medications used to treat smoking-related diseases (eg, buflomedil for peripheral arterial disease and angina pectoris, and insulin for insulin resistance of diabetes mellitus) with increased cancer risk (eg, congenital anomalies of the circulatory system [37] and periodontal conditions [38]), and paraneoplastic phenomena (eg, noninfectious gastroenteritis and colitis [39]). This information must be interpreted carefully, as these findings do not imply a causal relationship. For example, the model may predict an increased likelihood of future lung cancer in patients with pre-existing lung disease simply because these patients receive frequent medical attention; thus, there is a higher likelihood that cancer will be detected incidentally. In addition, the sensitivity analysis in this study is only capable of evaluating one factor at a time; this is a major limitation of the explainability of the model, given the fact that our model was designed to integrate complex, high-level features. Finally, we could not explain some of the predictive features identified by this model, such as the associations with terms including *antidote*, *orchitis*, and *epididymitis*. More studies will be required to decode the findings from the CNN and to elucidate the interactions between age, sex, previous diagnoses, and medications.

Although our model achieved excellent discriminative performance, poor calibration was noted, together with the fact that direct numeric output would overestimate the actual risk. This is a known phenomenon associated with modern neural networks [40]. Unlike the traditional logistic regression models, which perform well in calibration because they directly minimize the loss of calibration, modern neural networks tend to perform suboptimally in this regard. This is likely due to the regularization methods (eg, dropout and batch normalization) and the multiple deep layers applied as components of the model architecture [40]. In our study, poor calibration did not limit the use of the model, as individuals were selected based on a predefined threshold identified in the validation data set rather than on the numerical output of the model. As a result, the increased rates reported in Table 4 do not represent the actual cancer risk.

Our model used nonimaging medical information from EMRs; however, we still used CNN as the model backbone. The study design and aims are different from other lung cancer studies that used CNN to analyze computed tomography (CT) scans and determine if a pulmonary nodule is malignant. Their models were used to automatically identify suspicious nodules from CT scans, which were already present, whereas our model attempted to identify patients with high risk of developing lung cancer in the future.

### Limitations

There are several limitations to this study. First, the data collection was limited to the NHIRD database of Taiwan; the patient records do not include tissue histology or lung cancer staging data. Patients with small cell lung cancer and mutation-rich non–small cell lung cancer (eg, epidermal growth factor receptor, anaplastic lymphoma kinase, ROS-1) could not be separated. These specific types may have different disease courses and risk factors; therefore, they were usually not included in the traditional screening, and the benefit of receiving screening is undetermined. Our subgroup analysis did include only patients with pre-existing lung diseases, but this did not mitigate the issue entirely. Similarly, the NHIRD database does not include information on patients’ lifestyles or any genetic or laboratory data. A subgroup analysis of patients with lung cancer based on tissue histology and staging might help to develop a prediction model that was tailored to different risk groups. Second, the data set did not contain any information on smoking status, which is clearly an important risk factor associated with lung cancer development. This limitation restricted the external validation and the comparisons that could be made between our model and those described in earlier published studies. The authors believe that self-reported information, such as family history, smoking/cessation history, and duration of symptoms, are valuable pieces of information for lung cancer prediction that are very important and can further improve prediction accuracy. In our study, a history of lung diseases (eg, COPD and emphysema) was used as a proxy for a smoking history; our model performed with excellent discriminative power with respect to this subgroup. Finally, the NHIRD includes primarily Taiwanese people; as such, the target population was fairly homogeneous, with limited ethnic diversity. The identified risk factors may not apply to other populations with other ethnicities. Nonetheless, the methodology used here could be easily applied to other medical databases with more diverse patient populations.

### Conclusion

Our CNN model exhibited robust performance with respect to the 1-year prospective prediction of the risk of developing lung cancer. As our model included sequential data on clinical diagnoses and medication history, it was capable of capturing features associated with evolving clinical conditions and as such was able to identify patients at higher risk of developing lung cancer. With appropriate ethical regulation, this model may be deployed as a means to analyze medical databases, thus paving the way for efficient population-based screening and digital precision medicine. A future randomized controlled trial will be required to explore the clinical benefit of this model in diverse populations.

## Appendix

Multimedia Appendix 1 Supplementary tables and figures.

## Abbreviations

ATC: Anatomical Therapeutic Chemical
AUC: area under the receiver operating characteristic curve
CNN: convolutional neural network
COPD: chronic obstructive pulmonary disease
CT: computed tomography
EMR: electronic medical record
ICD-9-CM: International Classification of Diseases, Ninth Revision, Clinical Modification
LDCT: low-dose computed tomography
MOE: Ministry of Education
NHIRD: National Health Insurance Research Database
NLST: National Lung Cancer Screening Trial
PPV: positive predictive value
t-SNE: t-distributed stochastic neighbor embedding
WHO: World Health Organization
XGBoost: extreme gradient boosting
