# Correction: Artificial Intelligence–Based Prediction of Lung Cancer Risk Using Nonimaging Electronic Medical Records: Deep Learning Approach

**DOI:** 10.2196/33519

**Published:** 2021-10-15

**Authors:** Marvin Chia-Han Yeh, Yu-Hsiang Wang, Hsuan-Chia Yang, Kuan-Jen Bai, Hsiao-Han Wang, Yu-Chuan Jack Li

**Affiliations:** 1 Department of Dermatology Wan Fang Hospital Taipei Medical University Taipei Taiwan; 2 Research Center of Big Data and Meta-analysis Wan Fang Hospital Taipei Medical University Taipei Taiwan; 3 School of Medicine Taipei Medical University Taipei Taiwan; 4 Graduate Institute of Biomedical Informatics College of Medical Science and Technology Taipei Medical University Taipei Taiwan; 5 International Center for Health Information Technology Taipei Medical University Taipei Taiwan; 6 Division of Pulmonary Medicine, Department of Internal Medicine Wan Fang Hospital Taipei Medical University Taipei Taiwan; 7 School of Respiratory Therapy College of Medicine Taipei Medical University Taipei Taiwan; 8 Pulmonary Research Center Wan Fang Hospital Taipei Medical University Taipei Taiwan; 9 Department of Dermatology School of Medicine Taipei Medical University Taipei Taiwan

Correction: Artificial Intelligence–Based Prediction of Lung Cancer Risk Using Nonimaging Electronic Medical Records: Deep Learning Approach

In “Artificial Intelligence–Based Prediction of Lung Cancer Risk Using Nonimaging Electronic Medical Records: Deep Learning Approach” (J Med Internet Res 2021;23(8):e26256), two errors were noted.

Due to a system error, the name of one author, Marvin Chia-Han Yeh, was replaced with the name of another author on the paper, Hsuan-Chia Yang. As well, the formatting of the author name "Yu-Chuan (Jack) Li" has been changed to "Yu-Chuan Jack Li" in the corrected version of the paper.

In the originally published paper, the order of authors was listed as follows:

Hsuan-Chia Yang, Yu-Hsiang Wang, Hsuan-Chia Yang, Kuan-Jen Bai, Hsiao-Han Wang, Yu-Chuan (Jack) Li.

This has been corrected to:

Marvin Chia-Han Yeh, Yu-Hsiang Wang, Hsuan-Chia Yang, Kuan-Jen Bai, Hsiao-Han Wang, Yu-Chuan Jack Li.

In the originally published paper, the ORCID of author Hsuan-Chia Yang was incorrectly published as follows:

0000-0001-6710-4435

This has been corrected to:

0000-0001-9198-0697

The correction will appear in the online version of the paper on the JMIR Publications website on October 15, 2021, together with the publication of this correction notice. Because this was made after submission to PubMed, PubMed Central, and other full-text repositories, the corrected article has also been resubmitted to those repositories.

