# Supplementary material for: Artificial Intelligence–Based Prediction of Lung Cancer Risk Using Nonimaging Electronic Medical Records: Deep Learning Approach
Source: J Med Internet Res. 2021 Aug 3;23(8):e26256. doi: 10.2196/26256 (PMC8371476; doi:10.2196/26256)
Supplement: Multimedia Appendix 1 [file jmir_v23i8e26256_app1.docx]

**Artificial Intelligence to Predict Lung Cancer Risk with Electronic Medical Records: A Deep Learning and Big Data Approach**

Marvin Chia-Han Yeh, MD, PhD, Yu-Hsiang Wang, Hsuan-Chia Yang, PhD, Kuan-Jen Pai, MD , Hsiao-Hang Wang, MD, Yu-Chuan Li, MD, PhD

**Supplementary material**

Table S1 Demographics of Lung Cancer Patient and Control demographics

Table S2 Subgroup Demographics – 1:10 Age-and-gender-matched

Table S3 Subgroup Demographics – Patients Aged Above 55

Table S4 Subgroup Demographics – Patients Aged Below 55

Table S5 Subgroup Demographics – Patients with Lung Diseases

Table S6 Subgroup Demographics – Patients without Lung Diseases

Table S7 Subgroup Demographics – Patients Aged Above 55 with Lung Diseases

Table S8 Subgroup Demographics – Patients Aged Above 55 without Lung Diseases

Table S9 Subgroup Demographics – Patients Aged Below 55 with Lung Diseases

Table S10 Subgroup Demographics – Patients Aged Below 55 without Lung Diseases

Table S11 Demographics of lung cancer and control patients (full table)

Table S12. Model Input Performance Analysis

Table S13. The Predictions and Performances of the Best Model of Different Age Cut-offs

Table S14. Discrimination performance (testing set) of the model in the subgroups (full table)

Figure S1. Reliability curve of the model

S1. Model parameters and hyperparameters

S2.1 Receiver operating curves and prediction from different operating cutoffs – All patients

S2.2 Receiver operating curves and prediction from different operating cutoffs – Patients Aged Above 55

S2.3 Receiver operating curves and prediction from different operating cutoffs – Patients Aged Below 55

S2.4 Receiver operating curves and prediction from different operating cutoffs – Patients with Lung Diseases

S2.5 Receiver operating curves and prediction from different operating cutoffs – Patients without Lung Diseases

S2.6 Receiver operating curves and prediction from different operating cutoffs – Patients Aged Above 55 with Lung Diseases

S2.7 Receiver operating curves and prediction from different operating cutoffs – Patients Aged Above 55 without Lung Diseases

S2.8 Receiver operating curves and prediction from different operating cutoffs – Patients Aged Below 55 with Lung Diseases

S2.9 Receiver operating curves and prediction from different operating cutoffs – Patients Aged Below 55 without Lung Diseases

S3. F1-score, accuracy of the testing performance.

Table S1 Demographics of All Lung Cancer and Control Patients

|  |  | Lung cancer n. | | | Control n. | | |
| --- | --- | --- | --- | --- | --- | --- | --- |
|  |  | Male | Female | Total | Male | Female | Total |
| Age | | | | | | | |
|  | 20-30 | 54 | 52 | 106 | 135,669 | 153,663 | 289,332 |
|  | 30-40 | 161 | 204 | 365 | 147,406 | 164,971 | 312,377 |
|  | 40-50 | 537 | 422 | 959 | 146,010 | 153,585 | 299,595 |
|  | 50-60 | 1,118 | 937 | 2,055 | 121,335 | 126,269 | 247,604 |
|  | 60-70 | 1,535 | 1,076 | 2,611 | 66,634 | 72,693 | 139,327 |
|  | 70-80 | 1,920 | 1,217 | 3,137 | 44,121 | 48,024 | 92,145 |
|  | 80-90 | 1,606 | 778 | 2,384 | 22,200 | 20,574 | 42,774 |
| Lung Diseases | | | | | | | |
|  | 0 | 4,687 | 3,365 | 8,052 | 598,305 | 642,751 | 1,241,056 |
|  | 1 | 1,513 | 951 | 2,464 | 67,752 | 79,261 | 147,013 |
|  | 2 | 585 | 309 | 894 | 14,944 | 15,491 | 30,435 |
|  | 3+ | 146 | 61 | 207 | 2,374 | 2,276 | 4,650 |
| Diagnosis counts in 3 years | | | | | | | |
|  | 0-25 | 1,220 | 442 | 1,662 | 277,415 | 178,994 | 456,409 |
|  | 25-50 | 1,059 | 662 | 1,721 | 161,991 | 185,757 | 347,748 |
|  | 50-75 | 886 | 669 | 1,555 | 81,949 | 125,319 | 207,268 |
|  | 75-100 | 704 | 593 | 1,297 | 48,618 | 79,081 | 127,699 |
|  | 100-150 | 1,156 | 845 | 2,001 | 52,675 | 84,737 | 137,412 |
|  | 150-200 | 665 | 586 | 1,251 | 26,049 | 39,559 | 65,608 |
|  | 200+ | 1,241 | 889 | 2,130 | 34,678 | 46,332 | 81,010 |
| Medication counts in 3 years | | | | | | | |
|  | 0-25 | 878 | 393 | 1,271 | 200,058 | 138,258 | 338,316 |
|  | 25-50 | 770 | 456 | 1,226 | 137,237 | 134,039 | 271,276 |
|  | 50-75 | 634 | 414 | 1,048 | 88,582 | 107,154 | 195,736 |
|  | 75-100 | 516 | 426 | 942 | 58,279 | 79,330 | 137,609 |
|  | 100-150 | 890 | 683 | 1,573 | 70,234 | 102,321 | 172,555 |
|  | 150-200 | 647 | 507 | 1,154 | 40,130 | 58,728 | 98,858 |
|  | 200+ | 2,596 | 1,807 | 4,403 | 88,855 | 119,949 | 208,804 |

Table S2 Subgroup Demographics – 1:10 Age-and-gender-matched

|  |  | Lung cancer n. | | | Control n. | | |
| --- | --- | --- | --- | --- | --- | --- | --- |
|  |  | Male | Female | Total | Male | Female | Total |
| Age | | | | | | | |
|  | 20-30 | 54 | 52 | 106 | 540 | 520 | 1,060 |
|  | 30-40 | 161 | 204 | 365 | 1,610 | 2,040 | 3,650 |
|  | 40-50 | 537 | 422 | 959 | 5,370 | 4,220 | 9,590 |
|  | 50-60 | 1,118 | 937 | 2,055 | 11,180 | 9,370 | 20,550 |
|  | 60-70 | 1,535 | 1,076 | 2,611 | 15,350 | 10,760 | 26,110 |
|  | 70-80 | 1,920 | 1,217 | 3,137 | 19,199 | 12,170 | 31,369 |
|  | 80-90 | 1,606 | 778 | 2,384 | 16,060 | 7,780 | 23,840 |
| Lung Diseases | | | | | | | |
|  | 0 | 4,687 | 3,365 | 8,052 | 52,041 | 36,777 | 88,818 |
|  | 1 | 1,513 | 951 | 2,464 | 12,089 | 7,556 | 19,645 |
|  | 2 | 585 | 309 | 894 | 4,317 | 2,158 | 6,475 |
|  | 3+ | 146 | 61 | 207 | 862 | 369 | 1,231 |
| Diagnosis counts in 3 years | | | | | | | |
|  | 25- | 1,220 | 442 | 1,662 | 13,353 | 6,508 | 19,861 |
|  | 25-50 | 1,059 | 662 | 1,721 | 10,489 | 7,161 | 17,650 |
|  | 50-75 | 886 | 669 | 1,555 | 8,360 | 6,241 | 14,601 |
|  | 75-100 | 704 | 593 | 1,297 | 7,056 | 5,357 | 12,413 |
|  | 100-150 | 1,156 | 845 | 2,001 | 10,627 | 8,123 | 18,750 |
|  | 150-200 | 665 | 586 | 1,251 | 6,971 | 5,344 | 12,315 |
|  | 200+ | 1,241 | 889 | 2,130 | 12,453 | 8,126 | 20,579 |
| Medication counts in 3 years | | | | | | | |
|  | 25- | 878 | 393 | 1,271 | 10,379 | 5,402 | 15,781 |
|  | 25-50 | 770 | 456 | 1,226 | 7,641 | 4,980 | 12,621 |
|  | 50-75 | 634 | 414 | 1,048 | 6,239 | 4,542 | 10,781 |
|  | 75-100 | 516 | 426 | 942 | 5,131 | 3,890 | 9,021 |
|  | 100-150 | 890 | 683 | 1,573 | 8,570 | 6,248 | 14,818 |
|  | 150-200 | 647 | 507 | 1,154 | 6,754 | 5,050 | 11,804 |
|  | 200+ | 2,596 | 1,807 | 4,403 | 24,595 | 16,748 | 41,343 |

Table S3 Subgroup Demographics – Patients Aged Above 55

|  |  | Lung cancer n. | | | Control n. | | |
| --- | --- | --- | --- | --- | --- | --- | --- |
|  |  | Male | Female | Total | Male | Female | Total |
| Age | | | | | | | |
|  | 20-30 | 0 | 0 | 0 | 0 | 0 | 0 |
|  | 30-40 | 0 | 0 | 0 | 0 | 0 | 0 |
|  | 40-50 | 0 | 0 | 0 | 0 | 0 | 0 |
|  | 50-60 | 612 | 517 | 1,129 | 54,164 | 56,642 | 110,806 |
|  | 60-70 | 1,535 | 1,076 | 2,611 | 66,634 | 72,693 | 139,327 |
|  | 70-80 | 1,920 | 1,217 | 3,137 | 44,121 | 48,024 | 92,145 |
|  | 80-90 | 1,606 | 778 | 2,384 | 22,200 | 20,574 | 42,774 |
| Lung Diseases | | | | | | | |
|  | 0 | 3,646 | 2,481 | 6,127 | 145,271 | 155,045 | 300,316 |
|  | 1 | 1,344 | 778 | 2,122 | 30,168 | 32,247 | 62,415 |
|  | 2 | 545 | 276 | 821 | 9,810 | 9,082 | 18,892 |
|  | 3+ | 138 | 53 | 191 | 1,870 | 155 | 2,025 |
| Diagnosis counts in 3 years | | | | | | | |
|  | 25- | 772 | 262 | 1,034 | 36,715 | 24,102 | 60,817 |
|  | 25-50 | 735 | 399 | 1,134 | 30,843 | 28,189 | 59,032 |
|  | 50-75 | 718 | 467 | 1,185 | 25,043 | 27,057 | 52,100 |
|  | 75-100 | 604 | 442 | 1,046 | 20,351 | 24,136 | 44,487 |
|  | 100-150 | 1,028 | 676 | 1,704 | 28,926 | 37,251 | 66,177 |
|  | 150-200 | 615 | 523 | 1,138 | 17,671 | 23,161 | 40,832 |
|  | 200+ | 1,201 | 819 | 2,020 | 27,570 | 34,037 | 61,607 |
| Medication counts in 3 years | | | | | | | |
|  | 25- | 558 | 237 | 795 | 29,291 | 20,732 | 50,023 |
|  | 25-50 | 493 | 269 | 762 | 21,761 | 19,056 | 40,817 |
|  | 50-75 | 466 | 255 | 721 | 17,989 | 18,370 | 36,359 |
|  | 75-100 | 406 | 279 | 685 | 15,245 | 16,555 | 31,800 |
|  | 100-150 | 756 | 510 | 1,266 | 24,634 | 28,419 | 53,053 |
|  | 150-200 | 556 | 398 | 954 | 18,687 | 22,584 | 41,271 |
|  | 200+ | 2,438 | 1,640 | 4,078 | 59,512 | 72,217 | 131,729 |

Table S4 Subgroup Demographics – Patients Aged Below 55

|  |  | Lung cancer n. | | | Control n. | | |
| --- | --- | --- | --- | --- | --- | --- | --- |
|  |  | Male | Female | Total | Male | Female | Total |
| Age | | | | | | | |
|  | 20-30 | 54 | 52 | 106 | 135,669 | 153,663 | 289,332 |
|  | 30-40 | 161 | 204 | 365 | 147,406 | 164,971 | 312,377 |
|  | 40-50 | 537 | 422 | 959 | 146,010 | 153,585 | 299,595 |
|  | 50-60 | 506 | 420 | 926 | 67,171 | 69,627 | 136,798 |
|  | 60-70 | 0 | 0 | 0 | 0 | 0 | 0 |
|  | 70-80 | 0 | 0 | 0 | 0 | 0 | 0 |
|  | 80-90 | 0 | 0 | 0 | 0 | 0 | 0 |
| Lung Diseases | | | | | | | |
|  | 0 | 1,041 | 884 | 1,925 | 453,034 | 487,706 | 940,740 |
|  | 1 | 169 | 173 | 342 | 37,584 | 47,014 | 84,598 |
|  | 2 | 40 | 33 | 73 | 5,134 | 6,409 | 11,543 |
|  | 3+ | 8 | 8 | 16 | 504 | 717 | 1,221 |
| Diagnosis counts in 3 years | | | | | | | |
|  | 25- | 448 | 180 | 628 | 240,700 | 154,892 | 395,592 |
|  | 25-50 | 324 | 263 | 587 | 131,148 | 157,568 | 288,716 |
|  | 50-75 | 168 | 202 | 370 | 56,906 | 98,262 | 155,168 |
|  | 75-100 | 100 | 151 | 251 | 28,267 | 54,945 | 83,212 |
|  | 100-150 | 128 | 169 | 297 | 23,749 | 47,486 | 71,235 |
|  | 150-200 | 50 | 63 | 113 | 8,378 | 16,398 | 24,776 |
|  | 200+ | 40 | 70 | 110 | 7,108 | 12,295 | 19,403 |
| Medication counts in 3 years | | | | | | | |
|  | 25- | 320 | 156 | 476 | 170,767 | 117,526 | 288,293 |
|  | 25-50 | 277 | 187 | 464 | 115,476 | 114,983 | 230,459 |
|  | 50-75 | 168 | 159 | 327 | 70,593 | 88,784 | 159,377 |
|  | 75-100 | 110 | 147 | 257 | 43,034 | 62,775 | 105,809 |
|  | 100-150 | 134 | 173 | 307 | 45,600 | 73,902 | 119,502 |
|  | 150-200 | 91 | 109 | 200 | 21,443 | 36,144 | 57,587 |
|  | 200+ | 158 | 167 | 325 | 29,343 | 47,732 | 77,075 |

Table S5 Subgroup Demographics – Patients with Lung Diseases

|  |  | Lung cancer n. | | | Control n. | | |
| --- | --- | --- | --- | --- | --- | --- | --- |
|  |  | Male | Female | Total | Male | Female | Total |
| Age | | | | | | | |
|  | 20-30 | 12 | 5 | 17 | 9,985 | 11,467 | 21,452 |
|  | 30-40 | 18 | 37 | 55 | 11,459 | 16,047 | 27,506 |
|  | 40-50 | 92 | 75 | 167 | 13,961 | 17,209 | 31,170 |
|  | 50-60 | 236 | 214 | 450 | 15,022 | 18,441 | 33,463 |
|  | 60-70 | 416 | 278 | 694 | 12,640 | 15,234 | 27,874 |
|  | 70-80 | 712 | 424 | 1,136 | 13,214 | 12,384 | 25,598 |
|  | 80-90 | 758 | 288 | 1,046 | 8,789 | 6,246 | 15,035 |
| Lung Diseases | | | | | | | |
|  | 0 | 0 | 0 | 0 | 0 | 0 | 0 |
|  | 1 | 1,513 | 951 | 2,464 | 67,752 | 79,261 | 147,013 |
|  | 2 | 585 | 309 | 894 | 14,944 | 15,491 | 30,435 |
|  | 3+ | 146 | 61 | 207 | 2,374 | 2,276 | 4,650 |
| Diagnosis counts in 3 years | | | | | | | |
|  | 25- | 106 | 21 | 127 | 10,263 | 5,580 | 15,843 |
|  | 25-50 | 203 | 92 | 295 | 16,195 | 15,034 | 31,229 |
|  | 50-75 | 243 | 128 | 371 | 12,592 | 15,919 | 28,511 |
|  | 75-100 | 212 | 159 | 371 | 9,529 | 13,308 | 22,837 |
|  | 100-150 | 450 | 247 | 697 | 12,923 | 18,582 | 31,505 |
|  | 150-200 | 312 | 200 | 512 | 8,211 | 11,010 | 19,221 |
|  | 200+ | 718 | 474 | 1,192 | 15,357 | 17,595 | 32,952 |
| Medication counts in 3 years | | | | | | | |
|  | 25- | 68 | 28 | 96 | 6,441 | 4,524 | 10,965 |
|  | 25-50 | 107 | 57 | 164 | 9,781 | 8,699 | 18,480 |
|  | 50-75 | 141 | 55 | 196 | 9,544 | 10,104 | 19,648 |
|  | 75-100 | 122 | 78 | 200 | 8,006 | 9,615 | 17,621 |
|  | 100-150 | 268 | 168 | 436 | 12,329 | 16,284 | 28,613 |
|  | 150-200 | 231 | 150 | 381 | 8,768 | 11,710 | 20,478 |
|  | 200+ | 1,307 | 785 | 2,092 | 30,201 | 36,092 | 66,293 |

Table S6 Subgroup Demographics – Patients without Lung Diseases

|  |  | Lung cancer n. | | | Control n. | | |
| --- | --- | --- | --- | --- | --- | --- | --- |
|  |  | Male | Female | Total | Male | Female | Total |
| Age | | | | | | | |
|  | 20-30 | 42 | 47 | 89 | 125,684 | 142,196 | 267,880 |
|  | 30-40 | 143 | 167 | 310 | 135,947 | 148,924 | 284,871 |
|  | 40-50 | 445 | 347 | 792 | 132,049 | 136,376 | 268,425 |
|  | 50-60 | 882 | 723 | 1,605 | 106,313 | 107,828 | 214,141 |
|  | 60-70 | 1,119 | 798 | 1,917 | 53,994 | 57,459 | 111,453 |
|  | 70-80 | 1,208 | 793 | 2,001 | 30,907 | 35,640 | 66,547 |
|  | 80-90 | 848 | 490 | 1,338 | 13,411 | 14,328 | 27,739 |
| Lung Diseases | | | | | | | |
|  | 0 | 4,687 | 3,365 | 8,052 | 598,305 | 642,751 | 1,241,056 |
|  | 1 | 0 | 0 | 0 | 0 | 0 | 0 |
|  | 2 | 0 | 0 | 0 | 0 | 0 | 0 |
|  | 3+ | 0 | 0 | 0 | 0 | 0 | 0 |
| Diagnosis counts in 3 years | | | | | | | |
|  | 25- | 1,114 | 421 | 1,535 | 267,152 | 173,414 | 440,566 |
|  | 25-50 | 856 | 570 | 1,426 | 145,796 | 170,723 | 316,519 |
|  | 50-75 | 643 | 541 | 1,184 | 69,357 | 109,400 | 178,757 |
|  | 75-100 | 492 | 434 | 926 | 39,089 | 65,773 | 104,862 |
|  | 100-150 | 706 | 598 | 1,304 | 39,752 | 66,155 | 105,907 |
|  | 150-200 | 353 | 386 | 739 | 17,838 | 28,549 | 46,387 |
|  | 200+ | 523 | 415 | 938 | 19,321 | 28,737 | 48,058 |
| Medication counts in 3 years | | | | | | | |
|  | 25- | 810 | 365 | 1,175 | 193,617 | 133,734 | 327,351 |
|  | 25-50 | 663 | 399 | 1,062 | 127,456 | 125,340 | 252,796 |
|  | 50-75 | 493 | 359 | 852 | 79,038 | 97,050 | 176,088 |
|  | 75-100 | 394 | 348 | 742 | 50,273 | 69,715 | 119,988 |
|  | 100-150 | 622 | 515 | 1,137 | 57,905 | 86,037 | 143,942 |
|  | 150-200 | 416 | 357 | 773 | 31,362 | 47,018 | 78,380 |
|  | 200+ | 1,289 | 1,022 | 2,311 | 58,654 | 83,857 | 142,511 |

Table S7 Subgroup Demographics – Patients Aged Above 55 with Lung Diseases

|  |  | Lung cancer n. | | | Control n. | | |
| --- | --- | --- | --- | --- | --- | --- | --- |
|  |  | Male | Female | Total | Male | Female | Total |
| Age | | | | | | | |
|  | 20-30 | 0 | 0 | 0 | 0 | 0 | 0 |
|  | 30-40 | 0 | 0 | 0 | 0 | 0 | 0 |
|  | 40-50 | 0 | 0 | 0 | 0 | 0 | 0 |
|  | 50-60 | 141 | 117 | 258 | 7,205 | 9,024 | 16,229 |
|  | 60-70 | 416 | 278 | 694 | 12,640 | 15,234 | 27,874 |
|  | 70-80 | 712 | 424 | 1,136 | 13,214 | 12,384 | 25,598 |
|  | 80-90 | 758 | 288 | 1,046 | 8,789 | 6,246 | 15,035 |
| Lung Diseases | | | | | | | |
|  | 0 | 0 | 0 | 0 | 0 | 0 | 0 |
|  | 1 | 1,344 | 778 | 2,122 | 30,168 | 32,247 | 62,415 |
|  | 2 | 545 | 276 | 821 | 9,810 | 9,082 | 18,892 |
|  | 3+ | 138 | 53 | 191 | 1,870 | 1,559 | 3,429 |
| Diagnosis counts in 3 years | | | | | | | |
|  | 25- | 71 | 14 | 85 | 1,879 | 1,035 | 2,914 |
|  | 25-50 | 155 | 62 | 217 | 3,909 | 3,112 | 7,021 |
|  | 50-75 | 209 | 93 | 302 | 4,409 | 4,192 | 8,601 |
|  | 75-100 | 186 | 121 | 307 | 4,470 | 4,686 | 9,156 |
|  | 100-150 | 410 | 204 | 614 | 7,915 | 9,140 | 17,055 |
|  | 150-200 | 296 | 179 | 475 | 6,074 | 7,007 | 13,081 |
|  | 200+ | 700 | 434 | 1,134 | 13,192 | 13,716 | 26,908 |
| Medication counts in 3 years | | | | | | | |
|  | 25- | 46 | 17 | 63 | 1,366 | 912 | 2,278 |
|  | 25-50 | 71 | 38 | 109 | 2,232 | 1,796 | 4,028 |
|  | 50-75 | 114 | 37 | 151 | 2,600 | 2,431 | 5,031 |
|  | 75-100 | 104 | 55 | 159 | 2,610 | 2,645 | 5,255 |
|  | 100-150 | 244 | 127 | 371 | 5,294 | 5,532 | 10,826 |
|  | 150-200 | 199 | 118 | 317 | 4,704 | 5,184 | 9,888 |
|  | 200+ | 1,249 | 715 | 1,964 | 23,042 | 24,388 | 47,430 |

Table S8 Subgroup Demographics – Patients Aged Above 55 without Lung Diseases

|  |  | Lung cancer n. | | | Control n. | | |
| --- | --- | --- | --- | --- | --- | --- | --- |
|  |  | Male | Female | Total | Male | Female | Total |
| Age | | | | | | | |
|  | 20-30 | 0 | 0 | 0 | 0 | 0 | 0 |
|  | 30-40 | 0 | 0 | 0 | 0 | 0 | 0 |
|  | 40-50 | 0 | 0 | 0 | 0 | 0 | 0 |
|  | 50-60 | 471 | 400 | 871 | 46,959 | 47,618 | 94,577 |
|  | 60-70 | 1,119 | 798 | 1,917 | 53,994 | 57,459 | 111,453 |
|  | 70-80 | 1,208 | 793 | 2,001 | 30,907 | 35,640 | 66,547 |
|  | 80-90 | 848 | 490 | 1,338 | 13,411 | 14,328 | 27,739 |
| Lung Diseases | | | | | | | |
|  | 0 | 3,646 | 2,481 | 6,127 | 145,271 | 155,045 | 300,316 |
|  | 1 | 0 | 0 | 0 | 0 | 0 | 0 |
|  | 2 | 0 | 0 | 0 | 0 | 0 | 0 |
|  | 3+ | 0 | 0 | 0 | 0 | 0 | 0 |
| Diagnosis counts in 3 years | | | | | | | |
|  | 25- | 701 | 248 | 949 | 34,836 | 23,067 | 57,903 |
|  | 25-50 | 580 | 337 | 917 | 26,934 | 25,077 | 52,011 |
|  | 50-75 | 509 | 374 | 883 | 20,634 | 22,865 | 43,499 |
|  | 75-100 | 418 | 321 | 739 | 15,881 | 19,450 | 35,331 |
|  | 100-150 | 618 | 472 | 1,090 | 21,011 | 28,111 | 49,122 |
|  | 150-200 | 319 | 344 | 663 | 11,597 | 16,154 | 27,751 |
|  | 200+ | 501 | 385 | 886 | 14,378 | 20,321 | 34,699 |
| Medication counts in 3 years | | | | | | | |
|  | 25- | 512 | 220 | 732 | 27,925 | 19,820 | 47,745 |
|  | 25-50 | 422 | 231 | 653 | 19,529 | 17,260 | 36,789 |
|  | 50-75 | 352 | 218 | 570 | 15,389 | 15,939 | 31,328 |
|  | 75-100 | 302 | 224 | 526 | 12,635 | 13,910 | 26,545 |
|  | 100-150 | 512 | 383 | 895 | 19,340 | 22,887 | 42,227 |
|  | 150-200 | 357 | 280 | 637 | 13,983 | 17,400 | 31,383 |
|  | 200+ | 1,189 | 925 | 2,114 | 36,470 | 47,829 | 84,299 |

Table S9 Subgroup Demographics – Patients Aged Below 55 with Lung Diseases

|  |  | Lung cancer n. | | | Control n. | | |
| --- | --- | --- | --- | --- | --- | --- | --- |
|  |  | Male | Female | Total | Male | Female | Total |
| Age | | | | | | | |
|  | 20-30 | 12 | 5 | 17 | 9,985 | 11,467 | 21,452 |
|  | 30-40 | 18 | 37 | 55 | 11,459 | 16,047 | 27,506 |
|  | 40-50 | 92 | 75 | 167 | 13,961 | 17,209 | 31,170 |
|  | 50-60 | 95 | 97 | 192 | 7,817 | 9,417 | 17,234 |
|  | 60-70 | 0 | 0 | 0 | 0 | 0 | 0 |
|  | 70-80 | 0 | 0 | 0 | 0 | 0 | 0 |
|  | 80-90 | 0 | 0 | 0 | 0 | 0 | 0 |
| Lung Diseases | | | | | | | |
|  | 0 | 0 | 0 | 0 | 0 | 0 | 0 |
|  | 1 | 169 | 173 | 342 | 37,584 | 47,014 | 84,598 |
|  | 2 | 40 | 33 | 73 | 5,134 | 6,409 | 11,543 |
|  | 3+ | 8 | 8 | 16 | 504 | 717 | 1,221 |
| Diagnosis counts in 3 years | | | | | | | |
|  | 25- | 35 | 7 | 42 | 8,384 | 4,545 | 12,929 |
|  | 25-50 | 48 | 30 | 78 | 12,286 | 11,922 | 24,208 |
|  | 50-75 | 34 | 35 | 69 | 8,183 | 11,727 | 19,910 |
|  | 75-100 | 26 | 38 | 64 | 5,059 | 8,622 | 13,681 |
|  | 100-150 | 40 | 43 | 83 | 5,008 | 9,442 | 14,450 |
|  | 150-200 | 16 | 21 | 37 | 2,137 | 4,003 | 6,140 |
|  | 200+ | 18 | 40 | 58 | 2,165 | 3,879 | 6,044 |
| Medication counts in 3 years | | | | | | | |
|  | 25- | 22 | 11 | 33 | 5,075 | 3,612 | 8,687 |
|  | 25-50 | 36 | 19 | 55 | 7,549 | 6,903 | 14,452 |
|  | 50-75 | 27 | 18 | 45 | 6,944 | 7,673 | 14,617 |
|  | 75-100 | 18 | 23 | 41 | 5,396 | 6,970 | 12,366 |
|  | 100-150 | 24 | 41 | 65 | 7,035 | 10,752 | 17,787 |
|  | 150-200 | 32 | 32 | 64 | 4,064 | 6,526 | 10,590 |
|  | 200+ | 58 | 70 | 128 | 7,159 | 11,704 | 18,863 |

Table S10 Subgroup Demographics – Patients Aged Below 55 without Lung Diseases

|  |  | Lung cancer n. | | | Control n. | | |
| --- | --- | --- | --- | --- | --- | --- | --- |
|  |  | Male | Female | Total | Male | Female | Total |
| Age | | | | | | | |
|  | 20-30 | 42 | 47 | 89 | 125,684 | 142,196 | 267,880 |
|  | 30-40 | 143 | 167 | 310 | 135,947 | 148,924 | 284,871 |
|  | 40-50 | 445 | 347 | 792 | 132,049 | 136,376 | 268,425 |
|  | 50-60 | 411 | 323 | 734 | 59,354 | 60,210 | 119,564 |
|  | 60-70 | 0 | 0 | 0 | 0 | 0 | 0 |
|  | 70-80 | 0 | 0 | 0 | 0 | 0 | 0 |
|  | 80-90 | 0 | 0 | 0 | 0 | 0 | 0 |
| Lung Diseases | | | | | | | |
|  | 0 | 1,041 | 884 | 1,925 | 453,034 | 487,706 | 940,740 |
|  | 1 | 0 | 0 | 0 | 0 | 0 | 0 |
|  | 2 | 0 | 0 | 0 | 0 | 0 | 0 |
|  | 3+ | 0 | 0 | 0 | 0 | 0 | 0 |
| Diagnosis counts in 3 years | | | | | | | |
|  | 25- | 413 | 173 | 586 | 232,316 | 150,347 | 382,663 |
|  | 25-50 | 276 | 233 | 509 | 118,862 | 145,646 | 264,508 |
|  | 50-75 | 134 | 167 | 301 | 48,723 | 86,535 | 135,258 |
|  | 75-100 | 74 | 113 | 187 | 23,208 | 46,323 | 69,531 |
|  | 100-150 | 88 | 126 | 214 | 18,741 | 38,044 | 56,785 |
|  | 150-200 | 34 | 42 | 76 | 6,241 | 12,395 | 18,636 |
|  | 200+ | 22 | 30 | 52 | 4,943 | 8,416 | 13,359 |
| Medication counts in 3 years | | | | | | | |
|  | 25- | 298 | 145 | 443 | 165,692 | 113,914 | 279,606 |
|  | 25-50 | 241 | 168 | 409 | 107,927 | 108,080 | 216,007 |
|  | 50-75 | 141 | 141 | 282 | 63,649 | 81,111 | 144,760 |
|  | 75-100 | 92 | 124 | 216 | 37,638 | 55,805 | 93,443 |
|  | 100-150 | 110 | 132 | 242 | 38,565 | 63,150 | 101,715 |
|  | 150-200 | 59 | 77 | 136 | 17,379 | 29,618 | 46,997 |
|  | 200+ | 100 | 97 | 197 | 22,184 | 36,028 | 58,212 |

Table S11 Demographics of lung cancer and control patients

|  |  | n | Mean age (SD) | Male gender | Mean diagnosis record counts (SD) | Mean medication record counts (SD) |
| --- | --- | --- | --- | --- | --- | --- |
| whole population | lung cancer | 116,17 | 66.62 (14.01) | 59.7% | 121.62(113.19) | 202.68(208.97) |
|  | control | 1,423,154 | 44.95 (16.32) | 48.0% | 66.09(76.60) | 105.99(135.54) |
| age-and-gender match (1:10) | lung cancer | 11,617 | 66.62 (14.01) | 59.7% | 121.62(113.19) | 202.68(208.97) |
|  | control | 116,169 | 66.62 (14.01) | 59.7% | 117.99(113.67) | 190.22(196.78) |
| age ≥ 55 years | lung cancer | 9,261 | 71.99 (9.46) | 61.3% | 135.12(116.31) | 227.81(218.12) |
|  | control | 385,052 | 66.57 (9.04) | 48.6% | 114.23(106.76) | 184.50(189.50) |
| age < 55 years | lung cancer | 2,356 | 45.50 (7.55) | 53.4% | 68.58(80.42) | 103.90(126.71) |
|  | control | 1,038,102 | 36.93 (9.85) | 47.8% | 48.23(51.36) | 76.87(93.45) |
| with history of lung disease history a | lung cancer | 3,565 | 70.79(12.73) | 63.0% | 175.12(134.36) | 297.56(245.55) |
|  | control | 182,098 | 53.01(18.09) | 46.7% | 125.17(114.53) | 204.85(204.66) |
| without history of lung disease history a | lung cancer | 8,052 | 64.77(14.16) | 58.2% | 97.94(93.08) | 160.67(174.80) |
|  | control | 1,270,651 | 43.77(15.70) | 48.2% | 57.42(64.94) | 91.48(115.23) |
| age ≥ 55 years and with lung disease history | lung cancer | 3,134 | 74.16(9.12) | 64.7% | 183.84(131.75) | 314.28(246.50) |
|  | control | 84,736 | 69.41(9.45) | 49.4% | 173.69(131.58) | 284.35(235.25) |
| age ≥ 55 years and without lung disease history | lung cancer | 6,127 | 70.88(9.44) | 60.0% | 110.20(98.65) | 183.58(187.22) |
|  | control | 30,0316 | 65.77(8.75) | 48.4% | 97.46(91.93) | 156.32(163.78) |
| age< 55 years and with lung disease history | lung cancer | 431 | 46.31(7.28) | 50.4% | 111.65(136.09) | 175.91(200.16) |
|  | control | 97,362 | 38.74(9.82) | 44.4% | 82.95(75.07) | 135.66(141.03) |
| age< 55 years and without lung disease history | lung cancer | 1,925 | 45.31(7.60) | 54.1% | 58.93(57.10) | 87.78(96.22) |
|  | control | 940,740 | 36.74(9.83) | 48.2% | 44.64(46.80) | 70.78(84.75) |

^a^lung diseases including included asbestosis, bronchiectasis, chronic bronchitis, chronic obstructive pulmonary disease, emphysema, fibrosis, pneumonia, sarcoidosis, silicosis, and tuberculosis.

Table S12. Model input performance analysis

|  | Validation AUC | Testing AUC | Testing sensitivity | Testing specificity |
| --- | --- | --- | --- | --- |
| Matching age and sex with medical and diagnosis history | 0.801 | 0.820 | 0.648 | 0.865 |
| Simplified binary diagnostic and medication history | 0.852 | 0.827 | 0.761 | 0.752 |
| Diagnostic history | 0.842 | 0.821 | 0.817 | 0.686 |
| Medication history | 0.902 | 0.894 | 0.805 | 0.825 |
| Diagnostic and medication history | 0.911 | 0.902 | 0.804 | 0.837 |

Table S13. Prediction and Performance of the Best Model of Different Age Cut-offs (Performance from the best model)

| Age | lung cancer n. | control n. | Testing AUC | Sensitivity | Specificity | True positives | True Negatives | False Positives | False Negatives | Positive Predictive Value | Negative Predictive Value |
| --- | --- | --- | --- | --- | --- | --- | --- | --- | --- | --- | --- |
| Above 20 | 1,304 | 138,640 | 0.902 | 0.785 | 0.862 | 1,023 | 119,479 | 19,161 | 281 | 5.07% | 99.77% |
| Above 25 | 1,301 | 126,695 | 0.894 | 0.760 | 0.875 | 989 | 110,826 | 15,869 | 312 | 5.87% | 99.72% |
| Above 30 | 1,295 | 114,327 | 0.888 | 0.764 | 0.862 | 989 | 98,494 | 15,833 | 306 | 5.88% | 99.69% |
| Above 35 | 1,276 | 99,228 | 0.882 | 0.774 | 0.841 | 988 | 83,494 | 15,734 | 288 | 5.91% | 99.66% |
| Above 40 | 1,249 | 84,593 | 0.878 | 0.785 | 0.818 | 981 | 69,184 | 15,409 | 268 | 5.99% | 99.61% |
| Above 45 | 1,201 | 70,877 | 0.875 | 0.776 | 0.816 | 932 | 57,815 | 13,062 | 269 | 6.66% | 99.54% |
| Above 50 | 1,154 | 57,187 | 0.866 | 0.686 | 0.891 | 792 | 50,970 | 6,217 | 362 | 11.30% | 99.29% |
| Above 55* | 1,046 | 43,328 | 0.871 | 0.716 | 0.871 | 749 | 37,727 | 5,601 | 297 | 11.80% | 99.22% |
| Above 60 | 918 | 30,708 | 0.863 | 0.740 | 0.838 | 679 | 25,726 | 4,982 | 239 | 11.99% | 99.08% |
| Above 65 | 736 | 20,393 | 0.865 | 0.707 | 0.873 | 520 | 17,795 | 2,598 | 216 | 16.68% | 98.80% |
| Above 70 | 590 | 14,151 | 0.861 | 0.659 | 0.904 | 389 | 12,788 | 1,363 | 201 | 22.20% | 98.45% |
| Above 75 | 426 | 8,904 | 0.833 | 0.638 | 0.890 | 272 | 7,922 | 982 | 154 | 21.69% | 98.09% |
| Above 80 | 243 | 4,858 | 0.819 | 0.630 | 0.869 | 153 | 4,223 | 635 | 90 | 19.42% | 97.91% |
| Above 85 | 90 | 1,879 | 0.791 | 0.600 | 0.824 | 54 | 1,548 | 331 | 36 | 14.03% | 97.73% |

*Recommended cut-off age for screening with the low dose computer tomography by USPSTF

Table S14. Discrimination performance (testing set) of the model in the subgroups. (full table)

|  | Lung cancer n. | control n. | validation AUC(SD) | Testing AUC(SD) | Testing sensitivity (SD) | Testing specificity (SD) | True positive  n (SD) | True negative  n (SD) | False positive n (SD) | False negative n (SD) | PPV (SD) | NPV (SD) |
| --- | --- | --- | --- | --- | --- | --- | --- | --- | --- | --- | --- | --- |
| whole population | 1,304 | 138,640 | 0.909  (0.002) | 0.898  (0.002) | 0.805  (0.015) | 0.825  (0.018) | 1,049.6  (20.2) | 114,444.8  (2,488.8) | 24,195.2  (2,488.8) | 254.4  (20.2) | 4.2%  (0.3%) | 99.8%  (0%) |
| matching age and gender | 1,304 | 13,040 | 0.797  (0.004) | 0.818  (0.005) | 0.647  (0.017) | 0.873  (0.023) | 832.8  (28.1) | 11,428.2  (349.4) | 1,611.8  (349.4) | 471.2  (28.1) | 34.6%  (0.4%) | 96.0%  (0.1%) |
| Age ≥ 55 years | 1,046 | 43,328 | 0.854  (0.005) | 0.869  (0.002) | 0.784  (0.011) | 0.785  (0.016) | 819.8  (11.9) | 34,018.8  (699.4) | 9,309.2  (699.4) | 226.2  (11.9) | 8.1%  (0.5%) | 99.3%  (0%) |
| Age < 55 years | 258 | 95,312 | 0.840  (0.010) | 0.815  (0.007) | 0.620  (0.080) | 0.838  (0.054) | 160.0  (20.6) | 79,870.4  (5,141.4) | 15,441.6  (5,141.4) | 98.0  (20.6) | 1.1%  (0.2%) | 99.9%  (0%) |
| with lung disease history | 361 | 16,596 | 0.899  (0.007) | **0.914**  **(0.003)** | 0.829  (0.021) | 0.816  (0.021) | 299.4  (7.7) | 13,547.8  (342.9) | 3,048.2  (342.9) | 61.6  (7.7) | 9.0%  (0.8%) | 0.995  (0.1%) |
| without lung disease history | 943 | 122,044 | 0.903  (0.004) | 0.887  (0.002) | 0.78  1(0.025) | 0.827  (0.026) | 736.8  (23.8) | 100,953.6  (3,233.3) | 21,090.4  (3,233.4) | 206.2  (23.8) | 3.4%  (0.5%) | 99.8%  (0.0%) |
| Age ≥ 55 years with a lung disease history | 318 | 8,184 | 0.853  (0.011) | 0.875  (0.005) | 0.755  (0.047) | 0.819  (0.044) | 240.0  (15.0) | 6,698.8  (363.8) | 1,485.2  (363.8) | 78.0  (15.0) | **14.3**  **(2.3%)** | 98.9%  (0.2%) |
| Age ≥ 55 years without a lung disease history | 728 | 35,144 | 0.850  (0.011) | 0.865  (0.003) | 0.775  (0.019) | 0.786  (0.018) | 564.2  (13.7) | 27,628.8  (646.1) | 7,515.2  (646.1) | 163.800  (13.) | 7.0%  (0.4%) | 99.4%  (0.0%) |
| Age <55 years with a lung disease history | 43 | 8,412 | 0.857  (0.029) | 0.909  (0.006) | 0.777  (0.054) | 0.891  (0.036) | 33.4  (2.3) | 7,497.0  (302.1) | 915.0  (302.1) | 9.6  (2.3) | 3.8  (1.0%) | 99.9%  (0.0%) |
| Age <55 years without a lung disease history | 215 | 86,900 | 0.834  (0.017) | 0.797  (0.008) | 0.533  (0.048) | 0.865  (0.026) | 114.6  (10.4) | 75,187.6  (2,283.7) | 11,712.4  (2,283.7) | 100.4  (10.4) | **1.0%**  **(0.2%)** | 99.9%  (0.0%) |

Figure S1. Reliability curve of the model


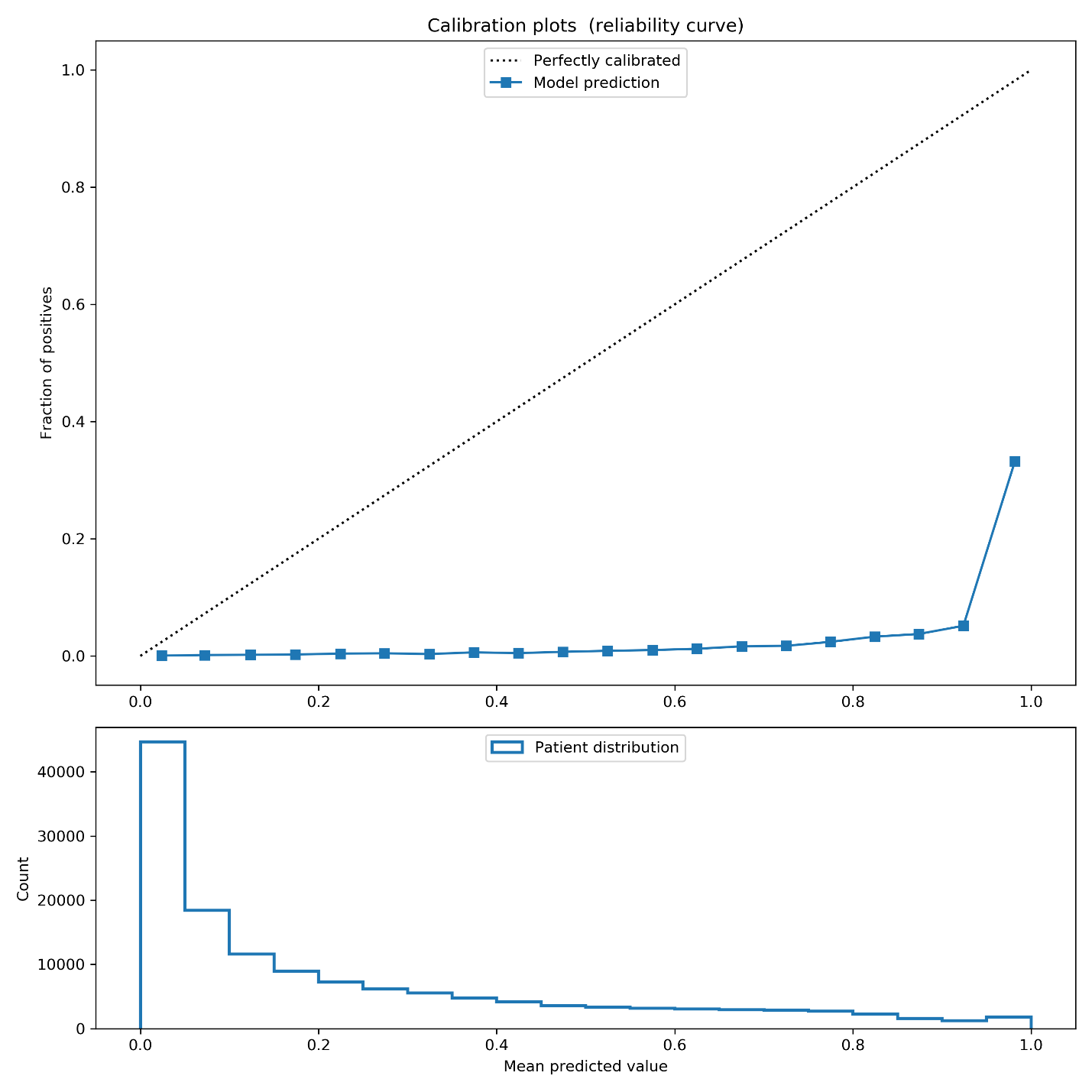


The reliability plot showed that the direct numeric outputs tend to over-estimate the actual risk. Therefore, the value should be used with a pre-defined threshold. The model showed a median absolute error of 0.125.

S1. Model training setting and hyperparameter

Machine learning backend: TensorFlow

Optimizer: Adam

Learning rate: 0.0005 (fixed)

Batch size: 32 patients data /GPU

Average pooling layer (avg) nodes: 2048

Fully connected layer (fc) nodes: 30

Activation function: relu

The Xception architecture was used but the parameters within the Xception was trained from scratch. The training was performed over a cloud computing service, TAIWANIA II, paralleled over eight GPUs (NVIDIA Tesla V100 32GB). The training of one model takes roughly 36 hours(~12 epochs).

S2.1 Receiver operating curves and prediction from different operating cutoffs – All patients


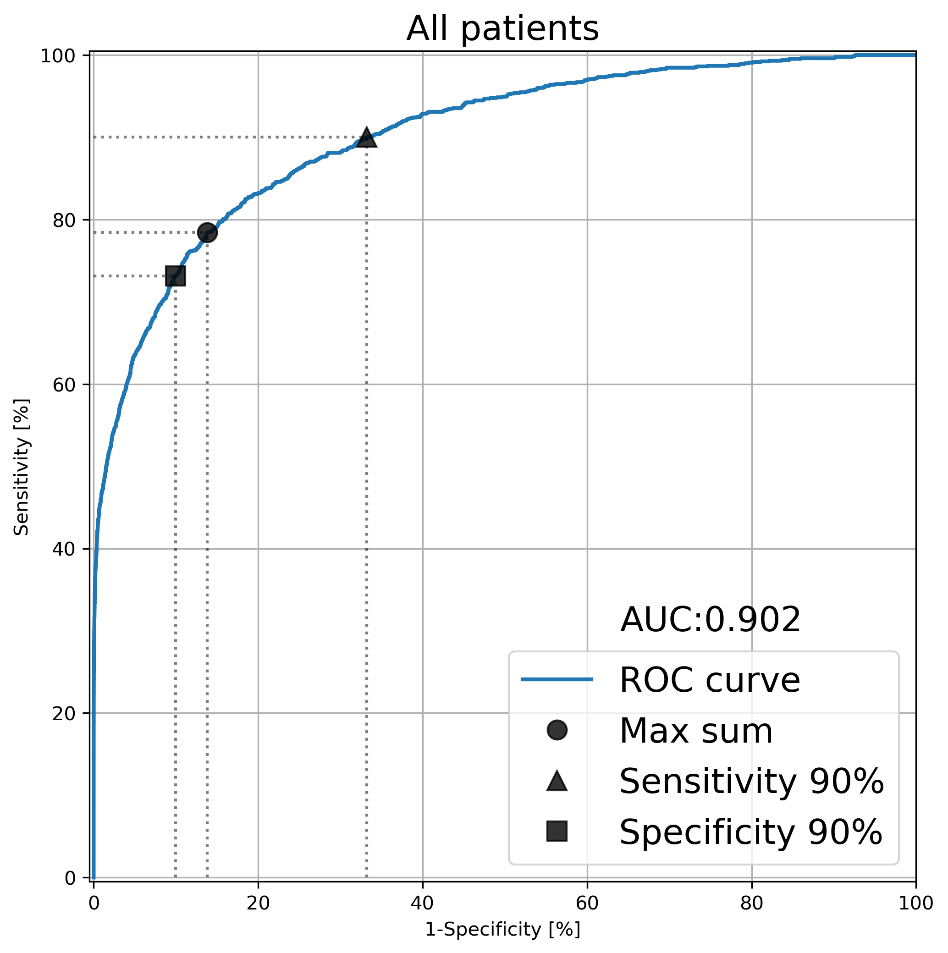


| Group | Lung Cancer n. | Control n. | AUC | Sensitivity | Specificity | True Positives | True Negatives | False Positives | False Negatives | Positive Predictive Value | Negative Predictive Value |
| --- | --- | --- | --- | --- | --- | --- | --- | --- | --- | --- | --- |
| All patients | 1,304 | 138,640 | 0.902 | 0.785 | 0.862 | 1,023 | 119,479 | 19,161 | 281 | 5.07% | 99.77% |
|  |  |  |  | 0.900 | 0.668 | 1,174 | 92,577 | 46,063 | 130 | 2.49% | 99.86% |
|  |  |  |  | 0.732 | 0.900 | 954 | 124,799 | 13,841 | 350 | 6.45% | 99.72% |

S2.2 Receiver operating curves and prediction from different operating cutoffs – Patients Aged Above 55


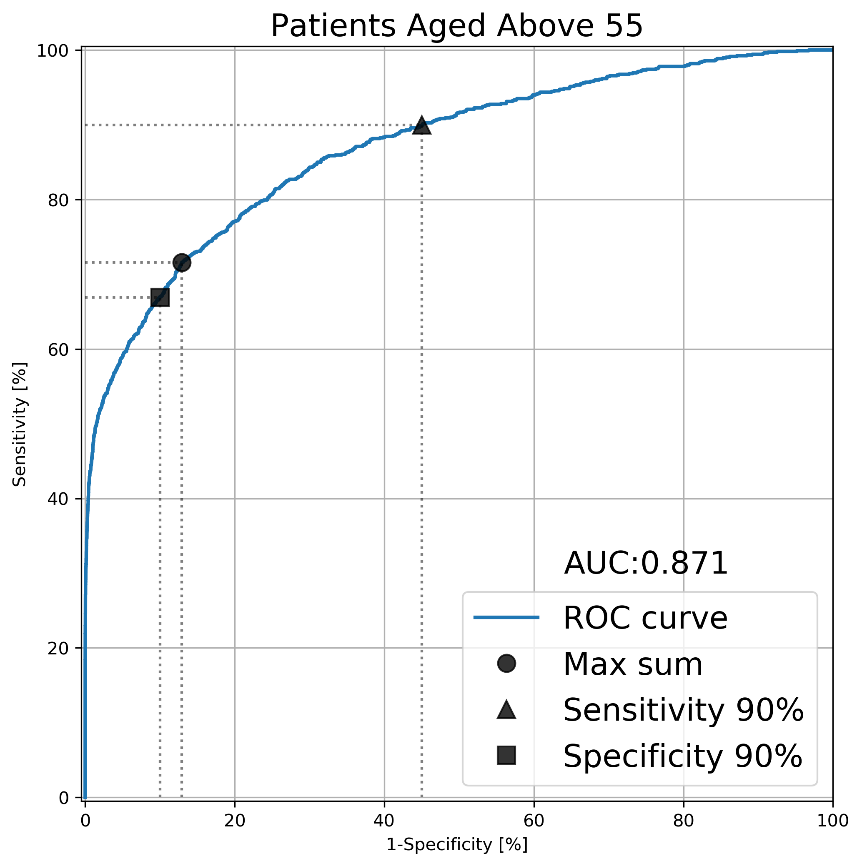


| Group | Lung Cancer n | Control n. | AUC | Sensitivity | Specificity | True Positives | True Negatives | False Positives | False Negatives | Positive Predictive Value | Negative Predictive Value |
| --- | --- | --- | --- | --- | --- | --- | --- | --- | --- | --- | --- |
| Age above 55 | 1,046 | 43,328 | 0.871 | 0.716 | 0.871 | 749 | 37,727 | 5,601 | 297 | 11.80% | 99.22% |
|  |  |  |  | 0.900 | 0.550 | 941 | 23,830 | 19,498 | 105 | 4.60% | 99.56% |
|  |  |  |  | 0.669 | 0.900 | 700 | 38,989 | 4,339 | 346 | 13.89% | 99.12% |

S2.3 Receiver operating curves and prediction from different operating cutoffs – Patients Aged Below 55


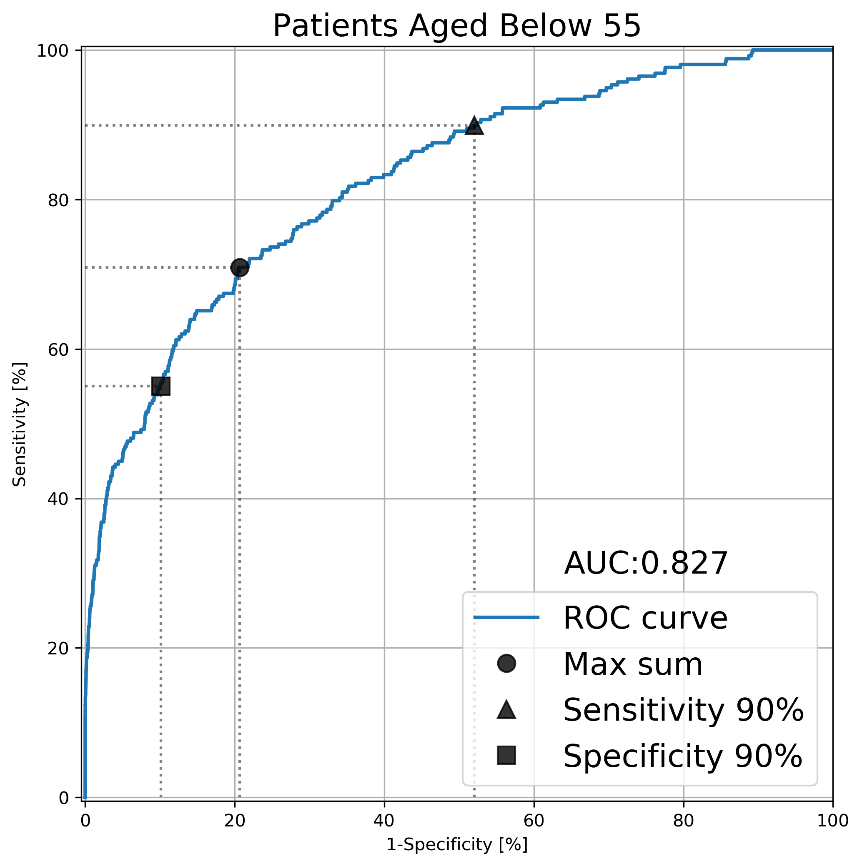


| Group | Lung Cancer n | Control n. | AUC | Sensitivity | Specificity | True Positives | True Negatives | False Positives | False Negatives | Positive Predictive Value | Negative Predictive Value |
| --- | --- | --- | --- | --- | --- | --- | --- | --- | --- | --- | --- |
| Age below 55 | 258 | 95,312 | 0.827 | 0.709 | 0.793 | 183 | 75,630 | 19,682 | 75 | 0.92% | 99.90% |
|  |  |  |  | 0.900 | 0.479 | 232 | 45,693 | 49,619 | 26 | 0.47% | 99.94% |
|  |  |  |  | 0.550 | 0.900 | 142 | 85,684 | 9,628 | 116 | 1.45% | 99.86% |

S2.4 Receiver operating curves and prediction from different operating cutoffs – Patients with Lung Diseases


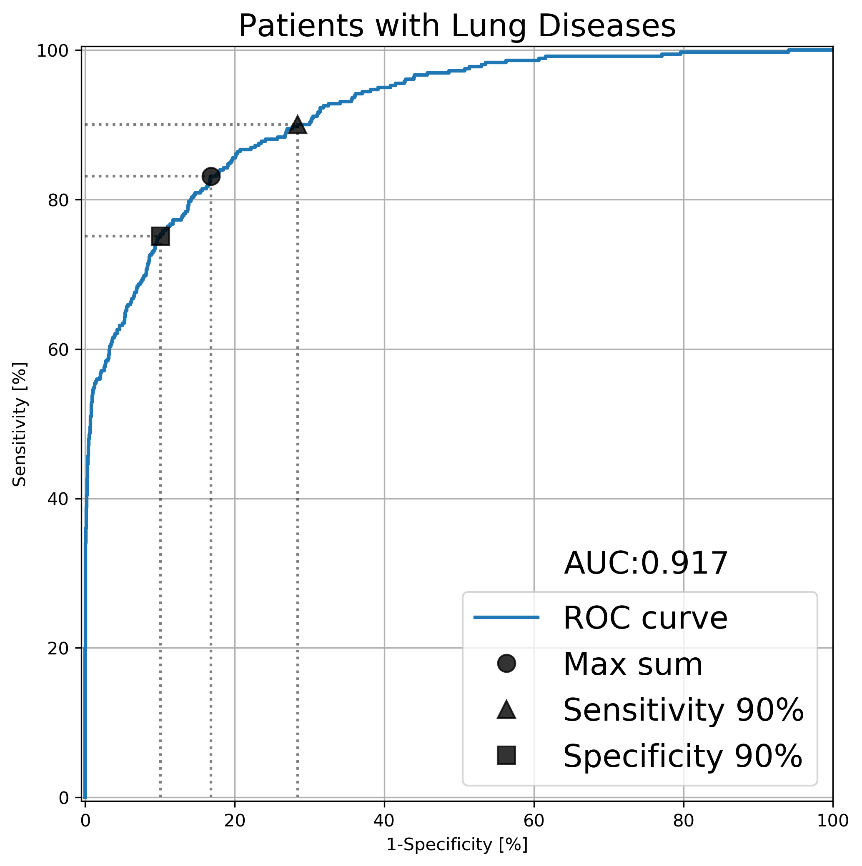


| Group | Lung Cancer n | Control n. | AUC | Sensitivity | Specificity | True Positives | True Negatives | False Positives | False Negatives | Positive Predictive Value | Negative Predictive Value |
| --- | --- | --- | --- | --- | --- | --- | --- | --- | --- | --- | --- |
| With Lung Diseases | 361 | 16,596 | 0.917 | 0.831 | 0.832 | 300 | 13,805 | 2,791 | 61 | 9.71% | 99.56% |
|  |  |  |  | 0.900 | 0.716 | 325 | 11,882 | 4,714 | 36 | 6.45% | 99.70% |
|  |  |  |  | 0.751 | 0.900 | 271 | 14,921 | 1,675 | 90 | 13.93% | 99.40% |

S2.5 Receiver operating curves and prediction from different operating cutoffs – Patients without Lung Diseases


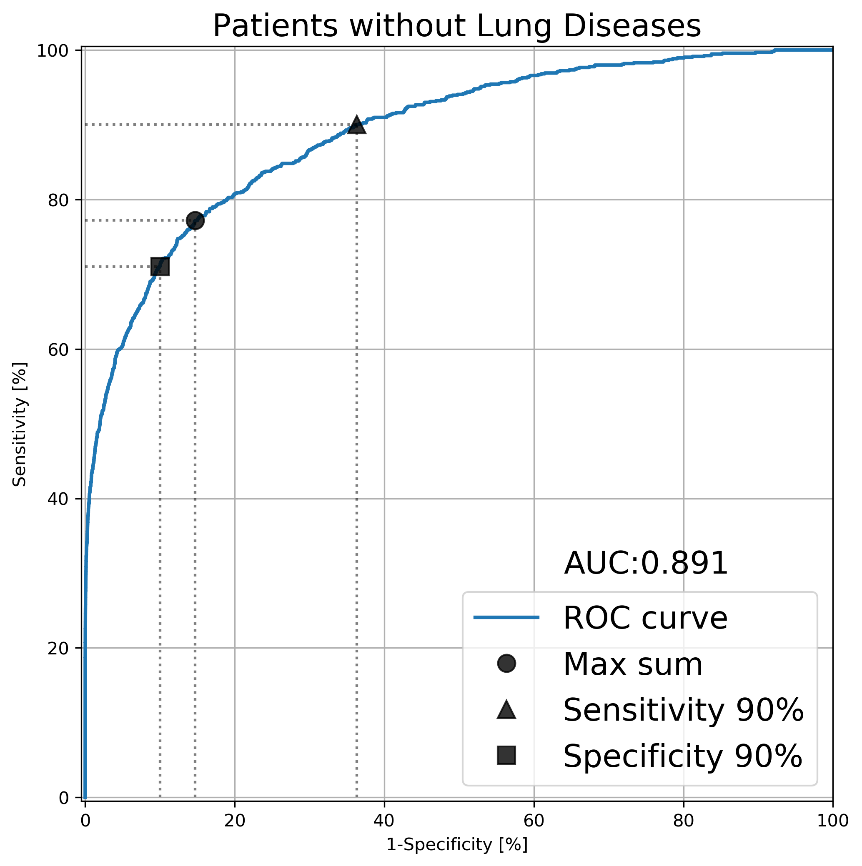


| Group | Lung Cancer n | Control n. | AUC | Sensitivity | Specificity | True Positives | True Negatives | False Positives | False Negatives | Positive Predictive Value | Negative Predictive Value |
| --- | --- | --- | --- | --- | --- | --- | --- | --- | --- | --- | --- |
| Without Lung Diseases | 943 | 122,044 | 0.891 | 0.772 | 0.853 | 728 | 104,077 | 17,967 | 215 | 3.89% | 99.79% |
|  |  |  |  | 0.900 | 0.637 | 849 | 77,737 | 44,307 | 94 | 1.88% | 99.88% |
|  |  |  |  | 0.710 | 0.900 | 670 | 109,829 | 12,215 | 273 | 5.20% | 99.75% |

S2.6 Receiver operating curves and prediction from different operating cutoffs – Patients Aged Above 55 with Lung Diseases


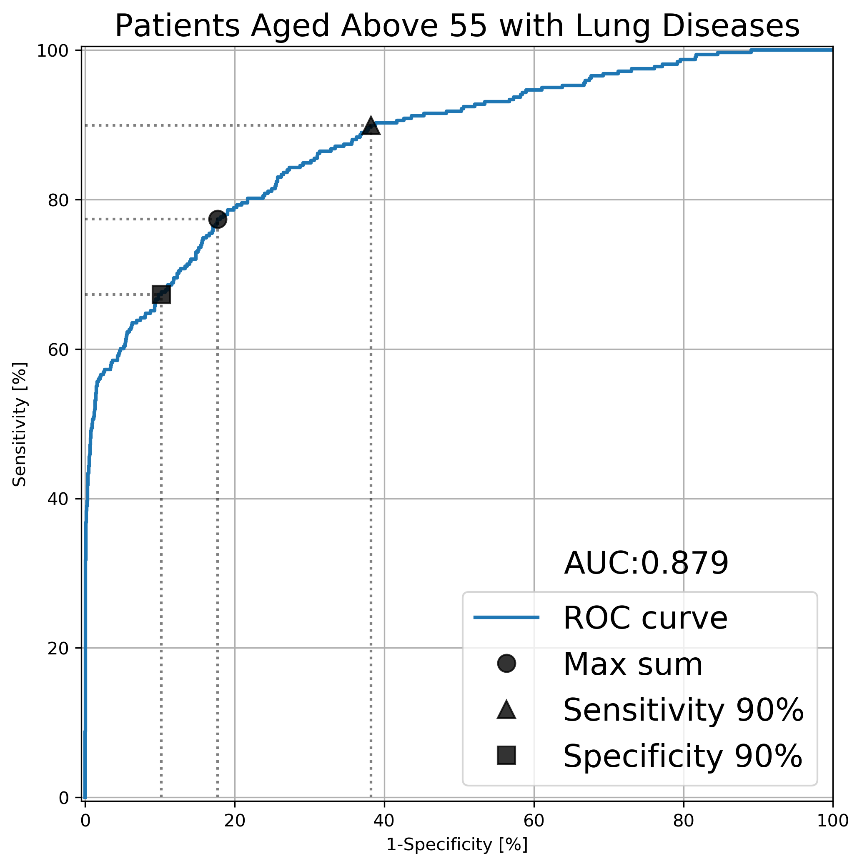


| Group | Lung Cancer n | Control n. | AUC | Sensitivity | Specificity | True Positives | True Negatives | False Positives | False Negatives | Positive Predictive Value | Negative Predictive Value |
| --- | --- | --- | --- | --- | --- | --- | --- | --- | --- | --- | --- |
| Aged Above 55 with Lung Diseases | 318 | 8,184 | 0.879 | 0.774 | 0.823 | 246 | 6,734 | 1,450 | 72 | 14.50% | 98.94% |
|  |  |  |  | 0.899 | 0.618 | 286 | 5,058 | 3,126 | 32 | 8.38% | 99.37% |
|  |  |  |  | 0.673 | 0.898 | 214 | 7,351 | 833 | 104 | 20.44% | 98.61% |

S2.7 Receiver operating curves and prediction from different operating cutoffs – Patients Aged Above 55 without Lung Diseases


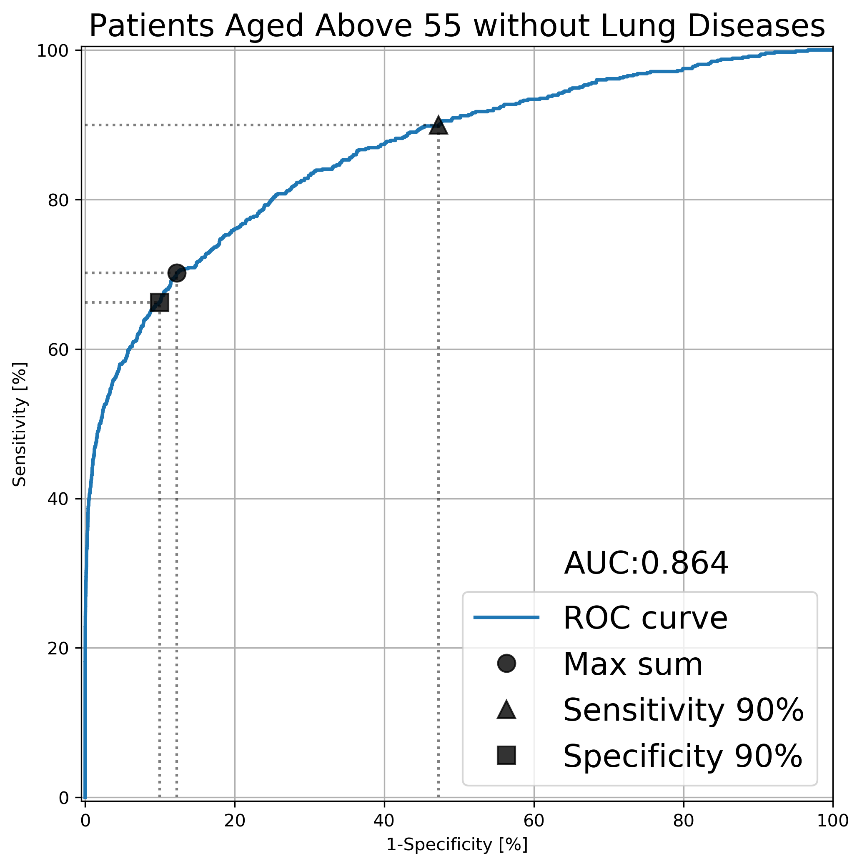


| Group | Lung Cancer n | Control n. | AUC | Sensitivity | Specificity | True Positives | True Negatives | False Positives | False Negatives | Positive Predictive Value | Negative Predictive Value |
| --- | --- | --- | --- | --- | --- | --- | --- | --- | --- | --- | --- |
| Aged Above 55 without Lung Diseases | 728 | 35,144 | 0.864 | 0.702 | 0.878 | 511 | 30,844 | 4,300 | 217 | 10.62% | 99.30% |
|  |  |  |  | 0.900 | 0.527 | 655 | 18,537 | 16,607 | 73 | 3.79% | 99.61% |
|  |  |  |  | 0.662 | 0.900 | 482 | 31,634 | 3,510 | 246 | 12.07% | 99.23% |

S2.8 Receiver operating curves and prediction from different operating cutoffs – Patients Aged Below 55 with Lung Diseases


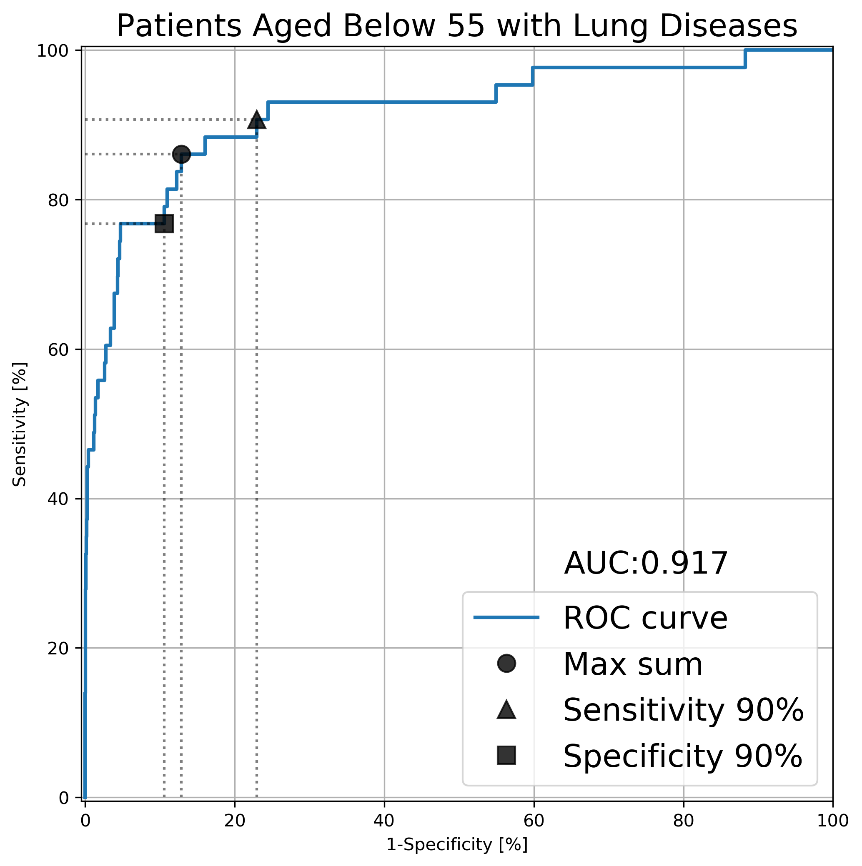


| Group | Lung Cancer n | Control n. | AUC | Sensitivity | Specificity | True Positives | True Negatives | False Positives | False Negatives | Positive Predictive Value | Negative Predictive Value |
| --- | --- | --- | --- | --- | --- | --- | --- | --- | --- | --- | --- |
| Aged Below 55 with Lung Diseases | 43 | 8,412 | 0.917 | 0.860 | 0.872 | 37 | 7,332 | 1,080 | 6 | 3.31% | 99.92% |
|  |  |  |  | 0.907 | 0.770 | 39 | 6,481 | 1,931 | 4 | 1.98% | 99.94% |
|  |  |  |  | 0.767 | 0.894 | 33 | 7,521 | 891 | 10 | 3.57% | 99.87% |

S2.9 Receiver operating curves and prediction from different operating cutoffs – Patients Aged Below 55 without Lung Diseases


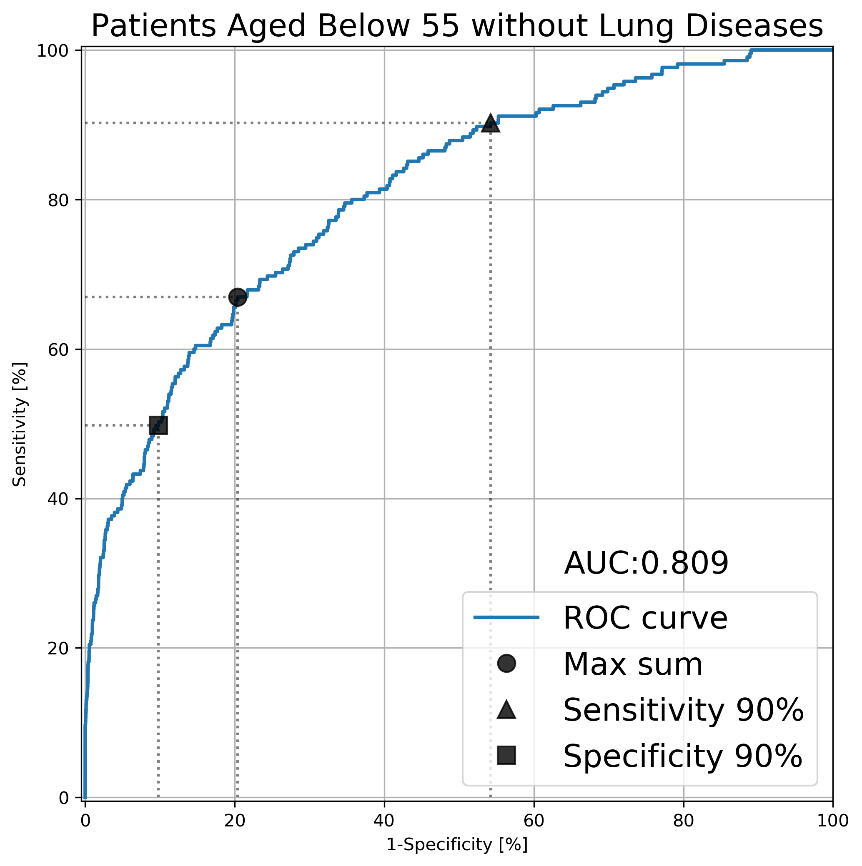


| Group | Lung Cancer n | Control n. | AUC | Sensitivity | Specificity | True Positives | True Negatives | False Positives | False Negatives | Positive Predictive Value | Negative Predictive Value |
| --- | --- | --- | --- | --- | --- | --- | --- | --- | --- | --- | --- |
| Aged Below 55 without Lung Diseases | 215 | 86,900 | 0.809 | 0.670 | 0.796 | 144 | 69,174 | 17,726 | 71 | 0.81% | 99.90% |
|  |  |  |  | 0.902 | 0.458 | 194 | 39,801 | 47,099 | 21 | 0.41% | 99.95% |
|  |  |  |  | 0.498 | 0.902 | 107 | 78,374 | 8,526 | 108 | 1.24% | 99.86% |

S3. F1-score, accuracy of the testing performance.

|  | Case n. | Control n. | Testing ROC | Accuracy | Recall | Precision | F1-score |
| --- | --- | --- | --- | --- | --- | --- | --- |
| whole population | 1,304 | 138,640 | 0.898(0.002) | 0.825(0.018) | 0.805(0.015) | 0.042(0.003) | 0.080(0.006) |
| age ≥ 55 years | 1,046 | 43,328 | 0.869(0.002) | 0.785(0.015) | 0.784(0.011) | 0.081(0.005) | 0.147(0.007) |
| age ≥ 55 years and with lung disease history | 318 | 8,184 | 0.875(0.005) | 0.816(0.041) | 0.755(0.047) | 0.143(0.023) | 0.239(0.030) |
| age ≥ 55 years and without lung disease history | 728 | 35,144 | 0.865(0.003) | 0.786(0.018) | 0.775(0.019) | 0.070(0.004) | 0.129(0.006) |
| age < 55 years | 258 | 95,312 | 0.815(0.007) | 0.837(0.054) | 0.620(0.080) | 0.011(0.002) | 0.021(0.005) |
| age< 55 years and with lung disease history | 43 | 8,412 | 0.909(0.006) | 0.891(0.035) | 0.777(0.054) | 0.038(0.010) | 0.072(0.018) |
| age< 55 years and without lung disease history | 215 | 86,900 | 0.797(0.008) | 0.864(0.026) | 0.533(0.048) | 0.010(0.002) | 0.019(0.003) |
| with history of lung disease history | 361 | 16,596 | 0.914(0.003) | 0.817(0.020) | 0.829(0.021) | 0.090(0.008) | 0.162(0.013) |
| without history of lung disease history | 943 | 122,044 | 0.887(0.002) | 0.827(0.026) | 0.781(0.025) | 0.034(0.005) | 0.066(0.008) |
